# Humoral Immunogenicity and Efficacy of a Single Dose of ChAdOx1 MERS Vaccine Candidate in Dromedary Camels

**DOI:** 10.1038/s41598-019-52730-4

**Published:** 2019-11-08

**Authors:** Naif Khalaf Alharbi, Ibrahim Qasim, Abdulrahman Almasoud, Haya A. Aljami, Mohamed W. Alenazi, Ali Alhafufi, Omar S. Aldibasi, Anwar M. Hashem, Samy Kasem, Raed Albrahim, Musaad Aldubaib, Ali Almansour, Nigel J. Temperton, Alexandra Kupke, Stephan Becker, Ali Abu-obaidah, Ali Alkarar, In-Kyu Yoon, Esam Azhar, Teresa Lambe, Faisal Bayoumi, Ali Aldowerij, Osman H. Ibrahim, Sarah C. Gilbert, Hanan H. Balkhy

**Affiliations:** 1 Department of Infectious Disease Research, King Abdullah International Medical Research Center, Riyadh, Saudi Arabia; 2 King Saud bin Abdulaziz University for Health Sciences, Riyadh, Saudi Arabia; 3 Ministry of Environment, Water and Agriculture (MEWA), Riyadh, Saudi Arabia; 4 Department of Bioinformatics and Biostatistics, King Abdullah International Medical Research Center, Riyadh, Saudi Arabia; 5 Department of Medical Microbiology and Parasitology, Faculty of Medicine, King Abdulaziz University, Jeddah, Saudi Arabia; 6 Vaccines and Immunotherapy Unit, King Fahd Medical Research Center, King Abdulaziz University, Jeddah, Saudi Arabia; 7 Department of Virology, Faculty of Veterinary Medicine, Kafrelsheikh University, Kafrelsheikh, El Geish Street, 33516 Egypt; 8 College of Agriculture and Veterinary Medicine, Qassim University, Qassim, Saudi Arabia; 9 Viral Pseudotype Unit, Medway School of Pharmacy, University of Kent, Chatham, Kent ME4 4TB United Kingdom; 10 Institute of Virology, Philipps University of Marburg, Marburg, Germany; 11 International Vaccine Institute, Seoul, South Korea; 12 German Center for Infection Research (DZIF), Partner Site Gieβen-Marburg-Langen, Germany; 13 Department of Medical Laboratory Technology, Faculty of Applied Medical Sciences, King Abdulaziz University, Jeddah, Saudi Arabia; 14 Special Infectious Agents Unit, King Fahd Medical Research Center, King Abdulaziz University, Jeddah, Saudi Arabia; 15 The Jenner Institute, University of Oxford, Oxford, OX3 7DQ UK; 16 Department of Infection Prevention and Control, Ministry of National Guard - Health Affairs, Riyadh, Saudi Arabia

**Keywords:** Vaccines, Viral transmission

## Abstract

MERS-CoV seronegative and seropositive camels received a single intramuscular dose of ChAdOx1 MERS, a replication-deficient adenoviral vectored vaccine expressing MERS-CoV spike protein, with further groups receiving control vaccinations. Infectious camels with active naturally acquired MERS-CoV infection, were co-housed with the vaccinated camels at a ratio of 1:2 (infected:vaccinated); nasal discharge and virus titres were monitored for 14 days. Overall, the vaccination reduced virus shedding and nasal discharge (p = 0.0059 and p = 0.0274, respectively). Antibody responses in seropositive camels were enhancedby the vaccine; these camels had a higher average age than seronegative. Older seronegative camels responded more strongly to vaccination than younger animals; and neutralising antibodies were detected in nasal swabs. Further work is required to optimise vaccine regimens for younger seronegative camels.

## Introduction

Middle East respiratory syndrome coronavirus (MERS-CoV) was first identified in 2012 from a pneumonic patient who subsequently died in Saudi Arabia. Since its emergence, the virus has infected more than 2200 individuals in 27 countries^1^. Outbreaks occurred mainly in the Arabian Peninsula in large crowded hospitals with one large outbreak in the Republic of Korea^2^. Although bats have been proposed as a possible natural reservoir of the virus^3^, dromedary camels are the only confirmed animal host so far. More than half (54.9%) of human primary cases have reported contact with camels^4^ and the index patient in the Korean outbreak travelled back from the Gulf countries where MERS-CoV is endemic and circulating in dromedary camels^5^. Several studies of camels showed 74–100% seroprevalence rates of MERS-CoV in Africa and the Arabian Peninsula^6–10^. The mean seroprevalence was higher in older camels than juvenile ones^9^. Furthermore, dromedary serum samples collected between 1983 and 2010 and archived in different countries in Africa and Arabia tested positive for MERS-CoV neutralising antibodies (nAbs)^11–16^. Moreover, a recent study in Saudi Arabia showed that 70% (n = 584) of dromedaries associated with confirmed human cases were seropositive with viral RNA being isolated from 12% of these dromedaries^5,17^. Importantly, sequencing the viral RNA from both patients and their camels confirmed genetic similarity in every paired case^5,17^. Therefore, it seems clear that MERS-CoV has been circulating in dromedaries in Africa and Arabia for the past three decades and has been causing human infections since 2012. Unlike the Arabian countries, there have been no reports of human cases in Africa, which could be due to under-diagnosis or differences in camel husbandry practices. One study has recently demonstrated that the virus in Africa may be genetically and phenotypically distinct^18^.

To date, there is no antiviral or vaccine to treat or prevent MERS-CoV in humans or camels. In camels, the MERS-CoV infection is mild, transient and does not require veterinary care. There is therefore little encouragement for camel owners to vaccinate their animals against MERS-CoV. However, there is an increasing body of evidence on asymptomatic and mildly symptomatic human cases amongst people who regularly have contact with camels, suggesting that these people may transmit the infection to the community^4,19^. Therefore, successful vaccination of camels would reduce virus circulation in this animal host and thereby reduce human exposure, leading to a reduction in the number of human cases. In addition, developing a vaccine for humans and vaccinating humans at risk, such as camel keepers and health care workers, would further control the number of human infections and prevent the spread of the virus to the general population. The same vaccine may be developed for use in both camels and humans.

The trimeric spike (S) glycoprotein is the main target of most experimental MERS-CoV vaccines as it plays an integral role in viral entry and fusion with target cells^20,21^. It consists of a receptor-binding domain (RBD)-containing S1 subunit that binds to dipeptidyl peptidase 4 (DPP4) on target cells, and a S2 subunit which contains the fusion peptide involved in viral fusion with target cells^20,21^. Several vaccine candidates have been developed and tested in multiple animal models including mice, rabbits, non-human primates and dromedaries (reviewed in^22–24^). While many of these vaccines were designed based on full-length S protein, some others were targeting the S1 subunit or the RBD of the protein to focus the immune response on the critical neutralising epitopes to induce nAbs. Nonetheless, non-neutralising antibodies (Abs) could also aid in protection against MERS-CoV by inhibiting essential steps in viral replication such as virus-target cell fusion. Furthermore, it has been shown that nAbs alone could not confer sterilising immunity but could provide partial protection in camels probably due to several factors including waning of nAbs and genetic change of viral lineage^25–29^. Thus, developing a camel vaccine based on full-length S protein could potentially elicit more robust antibody and T cell response compared to shorter targets.

We have developed a chimpanzee adenoviral vector based vaccine for MERS-CoV (ChAdOx1 MERS) that has now been tested in mouse models for immunogenicity^30^ and efficacy, demonstrating 100% efficacy after a single dose^31^. ChAdOx1 MERS was generated by inserting the full length of the spike gene, from a MERS-CoV isolate (Genbank accession number: KJ650098.1), into the genome of the replication deficient ChAdOx1 vector as described previously^30,32^. The ChAdOx1 viral vector of this vaccine is replication-deficient and has been assessed in different animal models, including dromedaries^33^, and in human clinical trials^34,35^. Here, we evaluated the immunogenicity and efficacy of ChAdOx1 MERS in dromedaries that are either seropositive or seronegative for MERS-CoV. We also evaluated the age effect on vaccine immunogenicity in seronegative dromedary calves. We have for the first time utilised a natural infection model of challenge to evaluate MERS vaccine efficacy in camels in a ‘real-world’ setting rather than a single exposure to MERS-CoV in a challenge experiment conducted within biocontainment facilities. This is also the first study to evaluate a MERS vaccine in dromedaries in endemic countries.

## Results

### Vaccination and challenge of dromedaries

**Figure 1:**
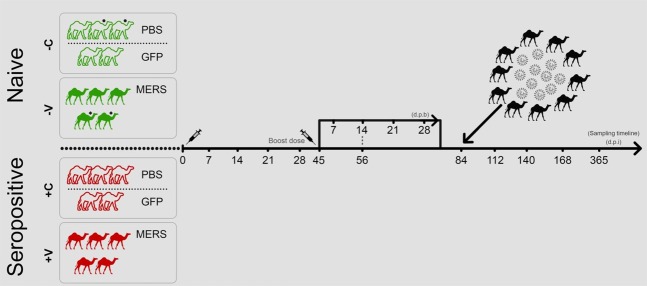
Evaluation of ChAdOx1 MERS vaccine in dromedary camels. Schematic diagram representing the study of immunogenicity and efficacy of ChAdOx1 MERS vaccine candidate in dromedary camels. +: Seropositive; −: seronegative; C: control group; V: vaccinated group. d.p.i.: days post immunisation. d.p.b.: days post boost. ● and ♦ indicate camels that were boosted with PBS or the vaccine, respectively, at 45 d.p.i.

Twenty calves (10 seronegative and 10 seropositive) were divided into four groups (n = 5/group). Group −C: seronegative camels receiving control injections of either PBS (n = 3) or ChAdOx1 encoding enhanced green fluorescent protein (ChAdOx1-eGFP) (n = 2), group −V: seronegative camels receiving ChAdOx1 MERS vaccine, group + C: seropositive camels receiving control injections of either PBS (n = 3) or ChAdOx1-eGFP (n = 2), and group + V: seropositive camels receiving ChAdOx1 MERS vaccine (Fig. 1 and Table 1). Serum samples were collected at day 0 pre-vaccination then at 7, 14, 21, 28, 56, 84, 112, 140, 168, and 365 days post immunisation (d.p.i.). In addition, at 45 d.p.i., two camels from groups −V or −C were boosted by a second injection of ChAdOx1 MERS or PBS, respectively; and samples were collected at 7, 14, 21, and 28 days post boost (d.p.b.) as shown in Fig. 1. Three months post prime (i.e. 84 d.p.i.), vaccinated camels were challenged in a natural infection model by introducing ten naturally infected and virus shedding camels, referred to as “infectious camels” in 1:2 (infectious:experimental) ratio, in order to evaluate the vaccine efficacy (Fig. 1). The MERS-CoV was tested in nasal swabs by RT-qPCR for 21 days post challenge (d.p.c.) and then at 28 and 56 d.p.c. Camels were all negative for MERS-CoV infection at 28 and 56 d.p.c., except one individual camel at 28 d.p.c. (Figure S4).

**Table 1:** Information on camels utilised in the main vaccination study.

Group	Camel #	Vaccine	Serology	Age	Gender	Boost vaccine
−C	1	PBS	Seronegative	6–12 m	F	PBS
	2	PBS	Seronegative	6–12 m	M	PBS
	3	PBS	Seronegative	6–12 m	F	None
	4	ChAdOx1-eGFP	Seronegative	6–12 m	M	None
	5	ChAdOx1-eGFP	Seronegative	6–12 m	M	None
−V	1	ChAdOx1 MERS	Seronegative	6–12 m	M	ChAdOx1 MERS
	2	ChAdOx1 MERS	Seronegative	6–12 m	M	None
	3	ChAdOx1 MERS	Seronegative	6–12 m	M	None
	4	ChAdOx1 MERS	Seronegative	6–12 m	M	None
	5	ChAdOx1 MERS	Seronegative	6–12 m	F	ChAdOx1 MERS
+C	1	ChAdOx1-eGFP	Seropositive	6–12 m	F	None
	2	PBS	Seropositive	18–24 m	F	None
	3	PBS	Seropositive	6–12 m	F	None
	4	ChAdOx1-eGFP	Seropositive	18–24 m	F	None
	5	PBS	Seropositive	18–24 m	M	None
+V	1	ChAdOx1 MERS	Seropositive	6–12 m	F	None
	2	ChAdOx1 MERS	Seropositive	24–30 m	F	None
	3	ChAdOx1 MERS	Seropositive	24–30 m	F	None
	4	ChAdOx1 MERS	Seropositive	24–30 m	F	None
	5	ChAdOx1 MERS	Seropositive	24–30 m	F	None

+: Seropositive.

−: Seronegative.

C: Control group.

V: Vaccinated group.

### ChAdOx1 MERS enhances antibody responses in seropositive dromedaries

**Figure 2:**
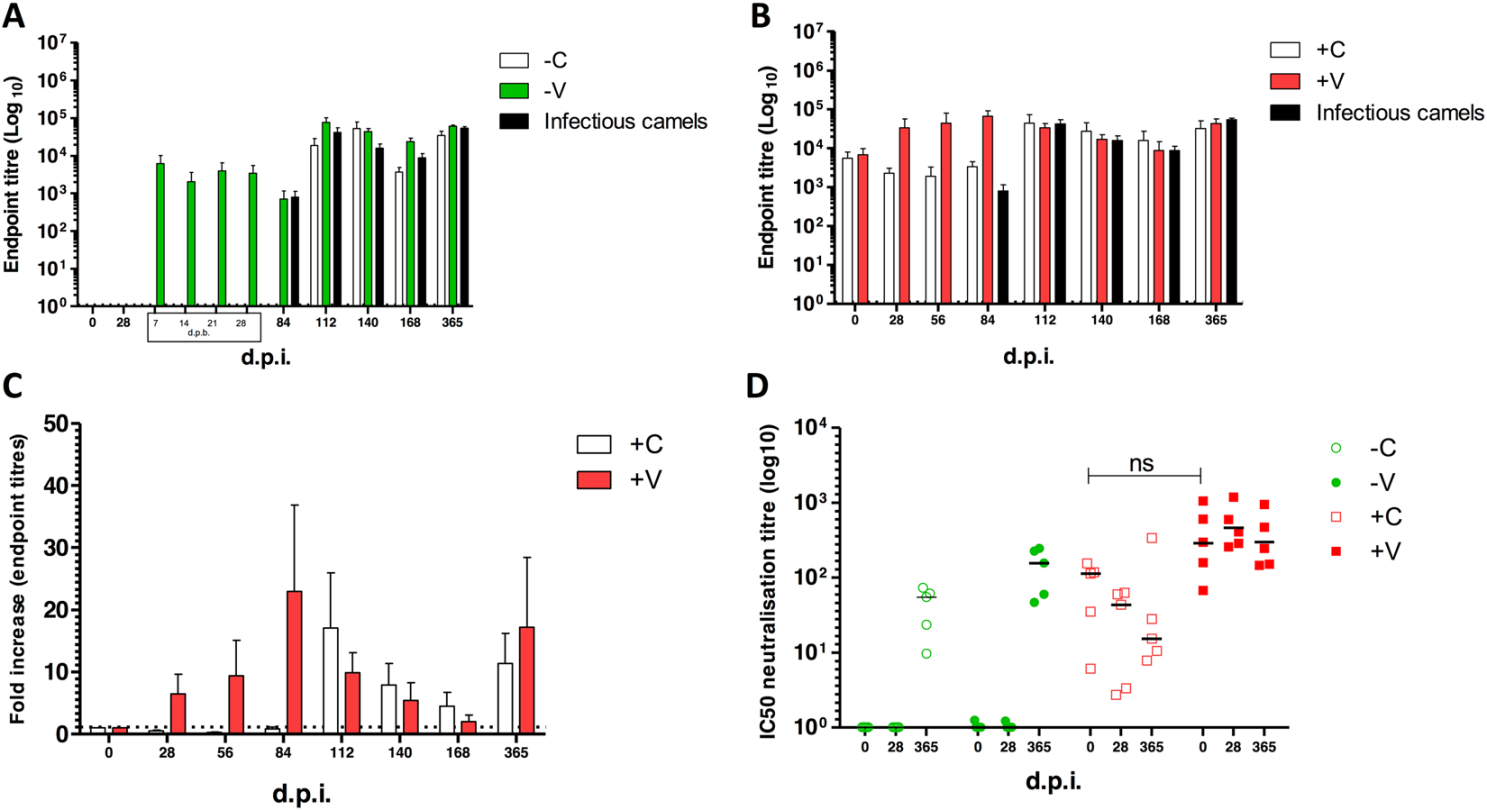
Antibody responses in dromedaries vaccinated with ChAdOx1 MERS. (**A**) Seronegative calves were either immunised with control injections (PBS or ChAdOx1 eGFP) in group −C (open bars), or with ChAdOx1 MERS in group −V (green bars). Two calves in group −V were boosted with a second dose of ChAdOx1 MERS at 45 d.p.i. Data in green bars prior to 84 d.p.i. are from the two boosted calves only; and data after 84 d.p.i. (after infection challenge) are from all five calves. (**B**) Seropositive camels were either immunised with control injections (PBS or ChAdOx1 eGFP) in group +C (open bars) or with ChAdOx1 MERS in group +V (red bars). Serum samples from different days (d.p.i. and d.p.b.) were evaluated for anti-S1 Ab endpoint titres. Infectious camels used as a natural challenge model (from 84 d.p.i.) were also evaluated (black bars) in (**A**,**B**). (**C**) The fold increase in Ab titres of groups +C and +V. (**D**) Analysis of nAbs in samples collected from 0, 28 and 365 d.p.i. (in **A**,**B**) using MERSpp NA. ns: No significant difference in antibody titres at day 0 in +C versus +V camels, by Kruskal-Wallis test with Dunn’s Multiple comparison test. +: Seropositive; −: seronegative; C: control group; V: vaccinated group. d.p.i.: days post immunisation. d.p.b.: days post boost.

Serum samples were evaluated using commercial ELISA kits (Figure S1) and an inhouse anti-S1 endpoint titre ELISA (Fig. 2). In seronegative camels (group −V), anti-S1 Abs were detectable only in the two camels that have received boost vaccination (Fig. 2A). These Ab levels were similar to the titres in naturally infected camels; however, these Ab titres decreased by 0.5 log by day 84 d.p.i., i.e. at the time of challenge (Fig. 2A). This indicates that generating measurable anti-spike Abs in naive dromedaries by vaccination is possible; however, these Abs decline over three months and vaccine boosting strategies should be considered for naive calves.

Conversely, vaccinating seropositive camels with ChAdOx1 MERS vaccine (group + V) enhanced S1-specific Ab titre by one log compared to group +C over the three months post vaccination (Fig. 2B,C and S1C), although both groups had similar baseline levels of Abs (0 d.p.i.). At the time of challenge, both groups had higher levels of S1-specific Abs compared to infectious camels. The fold increase in Ab endpoint titres in seropositive camels is shown in Fig. 2C.

After exposure to infectious camels, Ab levels increased in all groups, except in the + V group, which had the highest level of Abs pre-challenge. Overall, Ab levels in all groups plateaued for nine months post challenge up to 365 d.p.i. After the challenge infection, there was no substantial difference among seronegative camels in group −V that received either one or two doses of the vaccine, as indicated by small-range error bars in the Ab titres of −V group post challenge (data on 84 to 365 d.p.i., Fig. 2A).

Levels of nAbs were detected by a pseudotyped virus neutralisation assay (MERSpp NA) at 0, 28 and 365 d.p.i. As shown in Fig. 2D, only groups seropositive prior to vaccination (+C and +V) had nAbs at day 0, with no significant differences between these two groups. Vaccination did not increase these titres at 28 d.p.i.; however the titres in the vaccinated group (+V) were maintained at the same level on days 28 and 365, unlike the non-vaccinated camels that showed decreased nAb titres at 28 and 365 d.p.i.

### ChAdOx1 MERS significantly reduces rhinorrhoea in challenged dromedaries

**Figure 3:**
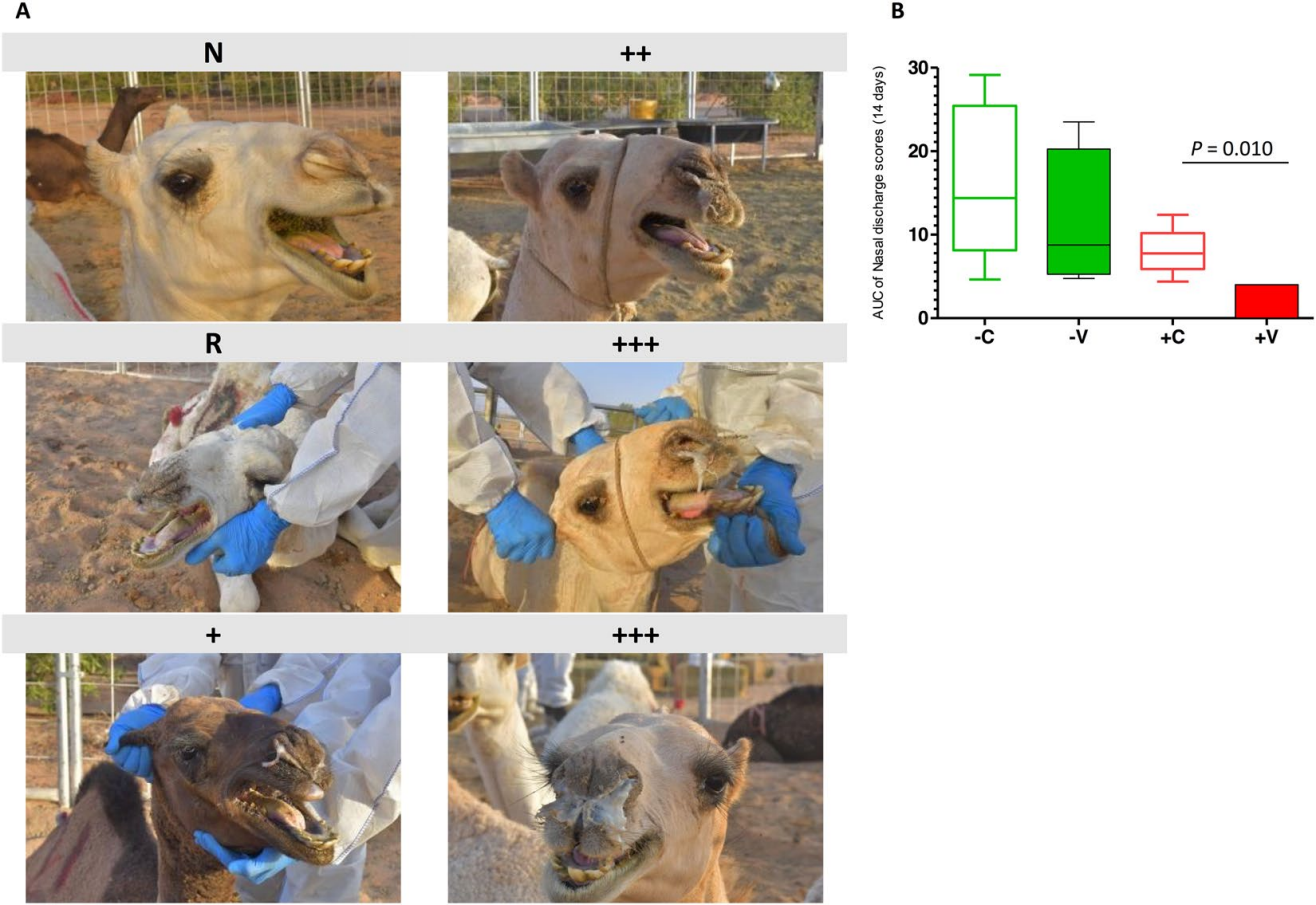
Severity and abundance of nasal discharge post natural infection challenge. The twenty camels in all groups (−C, −V, +C, and +V) were challenged with ten naturally infectious camels; and nasal discharges were assessed for their abundance and severity for 14 d.p.c.; using arbitrary units of 0 (N: normal), 0.25 (R: recovered), 1 (+: mild), 2 (++: moderate), or 3 (+++: severe). (**A**) Representative photos taken from different camels in different days. (**B**) Areas under the curve (AUC) for the nasal discharge scores collected daily from 1 to 14 d.p.c. are plotted. The analysis of mixed model on AUC of nasal discharge scores and fixed factors (d.p.c., seropositivity, and vaccine) shows that the overall vaccine effect over time was significant, p = 0.0274.; also +V had statistically significantly lower scores as compared to +C, by Kruskal-Wallis test with Dunn’s Multiple comparison test. d.p.i.: days post immunisation. +: Seropositive; −: seronegative; (**C**) control group; V: vaccinated group.

Ten infectious camels (MERS-CoV naturally infected and viral shedding camels) confirmed to be positive for MERS-CoV by RT-qPCR with a Ct value below 30 (Figure S2) were co-housed with the experimental 20 calves from the vaccination experiment in one pen starting from 84 d.p.i. (Fig. 1), resulting in a total of 30 camels in a pen of 30 m2. As an arbitrary indication of infection, nasal discharges from camels in all groups were evaluated and scored from 0–3 (from normal to severe; as explained in the methods) depending on the discharge abundance for 14 d.p.c. Figure S3. Areas under the curve (AUC) of these scores were plotted in Fig. 3. Most camels in seropositive vaccinated (+V) and control (+C) groups had mild nasal discharges that resolved 5 and 7 days post challenge, respectively (Figure S3B,D); only three camels in both seropositive groups had severe nasal discharge that lasted for one or two days only. The seronegative groups (−C and −V) showed nasal discharge that persisted for two weeks. Overall, the vaccine in both seropositive and seronegative camels reduced the nasal discharge over time significantly as compared to control camels, p = 0.0274 as analysed by mixed model analysis. In the group −V, the vaccine effect on reducing nasal discharges was not substantially different between prime-boost vaccinated camels (camel number: −V-01 and −V-05) and prime-only vaccinated camels (Figure S3C). The areas under the curve (AUC) of nasal discharge scores were also plotted to show the overall reduction in these scores in vaccinated camels as compared to the control camels, seropositive vaccinated camels had significant overall reduction in nasal discharge, *p* = 0.01 (Fig. 3B).

### ChAdOx1 MERS effectively minimises viral shedding in vaccinated dromedaries

Nasal swabs from all camels were collected daily for 21 days following the introduction of the infectious camels (challenge day) and then at 28 and 56 post challenge. Interestingly, the total number of infectious and virus shedding camels in the pen increased during the challenge study and varied over the weeks post challenge (Figure S4A). All of the 5 seronegative control (−C) camels as well as some camels from other groups were infected very early and the number of virus shedding camels peaked to 23 camels by the second day post challenge, resulting in a very high viral exposure for uninfected camels. The number of infected camels remained >18 during the first week and started to decline by the second week post challenge (Figure S4A).

**Figure 4:**
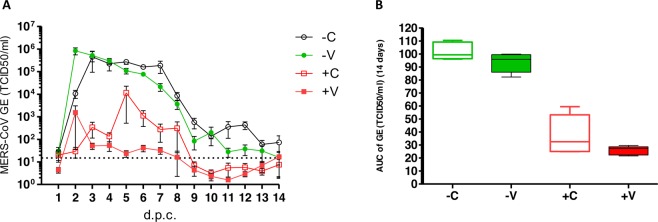
Efficacy of ChAdOx1 MERS in dromedaries based on GE TCID_50_/ml. Nasal swab samples from 1 to 14 days post challenge (d.p.c.) were assessed in absolute RT-qPCR quantification and the MERS-CoV viral genome equivalent (GE) to TCID_50_/ml is reported. (**A**) The mean titres of MERS-CoV GE (TCID_50_/ml) are reported from 1 to 14 days post challenge (d.p.c.) with SEM error bars for each group. (**B**) Areas under the curve (AUC) for the mean MERS-CoV GE (TCID_50_/ml) titres measured from 1 to 14 d.p.c. are plotted for each group. +: Seropositive; −: seronegative; (**C**) control group; V: vaccinated group. The analysis of mixed model on the log-transformed MERS-CoV GE (TCID_50_/ml) titres and fixed factors (d.p.c., seropositivity, and vaccine) shows that the vaccine was significant with p = 0.0059.

Viral shedding was evaluated by testing for MERS-CoV RNA and evaluating viral titre in all post challenge nasal swabs using RT-qPCR (Fig. 4 and S4). In all groups, viral RNA decreased to undetectable levels by 14 post challenge (Figure S4G). Seronegative vaccinated and control camels (−V and −C) shed high levels of the virus starting from day 2 to 9 post-challenge. On the contrary, seropositive camels (+C and +V) shed low levels of the virus post challenge (Fig. 4A). Of note, vaccinated seronegative and seropositive camels (−V and +V) had ~2 log10 higher virus titres (TCID_50_eq/ml) at day 2 post challenge as compared to the control seronegative and seropositive camels (−C and +C). However, virus titres in the vaccinated camels (−V and +V groups) rapidly decreased over time as compared to the control camels (−C and +C) (Fig. 4A). Infectious camels had high titres of viral RNA starting from the day of challenge (day 0, measured before housing with the study camels) and declining over time (Figures S2A and S4F).

There was no significant difference in viral shedding between seronegative camels receiving a single or two doses of ChAdOx1 MERS (as seen by small range error bars in data of −V group, Fig. 4A) despite camels receiving prime-boost immunisation had significant levels of anti-S1 Abs, which is targeting the main antigen that facilitates viral entry. Vaccine intervention over time (analysing the vaccine effect on virus titres in all days from 1 to 14) was statistically significant, p = 0.0059, in all vaccinated camels (−V and +V groups), as analysed by mixed model analysis and as shown by the lower viral shedding in vaccinated camels compared to controls especially in seropositive camels (Fig. 4A). Areas under the curve (AUC) of the average virus titres in each group was also reported (Fig. 4B).

### Single dose of ChAdOx1 MERS induces immune responses in old seronegative dromedary calves

**Figure 5:**
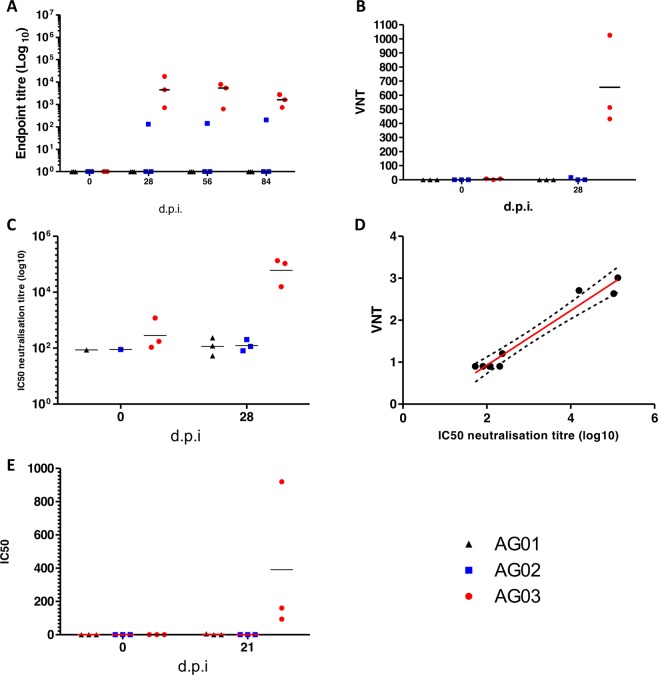
Antibody immune responses to ChAdOx1 MERS in dromedary calves at different ages. Seronegative dromedary calves, below 1 year old, were immunised with PBS (n = 2) or ChAdOx1-eGFP (n = 1) in group AG01 (black triangles) or with ChAdOx1 MERS in group AG02 (n = 3; blue squares). Older calves, 1–2 years old, were immunised with ChAdOx1 MERS in group AG03 (n = 3; red circles). (**A**) Anti-spike Ab titres were assessed in ELISA; and nAb titres were determined in MERS-CoV neutralisation assay (**B**) and in MERS pseudotyped virus neutralisation assay (MERSpp NA) (**C**). (**D**) Linear regression between the values of MERS-CoV neutralisation assay and MERSpp NA showed a significant relationship with R^2^ = 0.96, p < 0.0001. (**A**) Nasal mucosal nAbs were determined by MERSpp NA in samples from 0 and 21 d.p.c.

In a separate attempt to evaluate the effect of age on vaccine immunogenicity in dromedary calves, nine seronegative calves were vaccinated (n = 3/group) in a separate study. Group AG01 acted as control group with calves younger than one year receiving PBS (n = 2) or ChAdOx1-eGFP (n = 1), group AG02 with calves younger than one year receiving a single dose of ChAdOx1 MERS, and group AG03 with calves aged between one and two years receiving a single dose of ChAdOx1 MERS. Serum samples were collected at day 0 pre-vaccination, and 7, 14, 21, 28, 56, and 84 d.p.i., and nasal swabs were also collected at 0, and 21 d.p.i. No detectable anti-S1 Abs were induced in the younger calves in AG02 group except for one camel with very low level of Abs, whereas Abs were detected in the older calves in group AG03 (Fig. 5A and S5). Similarly, circulatory and local nAb were only induced in older claves in group AG03 but not those in group AG02 (Fig. 5B–E). Interestingly, the pseudotyped virus neutralisation assay (MERSpp NA) correlated significantly with the MERS-CoV neutralisation assay, R^2^ = 0.96, *p* < 0.0001. (Fig. 5D), both applied on same sera.

Subsequent to vaccination and initial sampling, all calves in the age group study were kept for serological follow up in their pen, which is a separate pen, 200 m away from the pen of the main study, in which we performed the natural challenge. Unexpectedly, all calves in the age group study (including the control calves) demonstrated immune responses as assessed by two ELISAs (Figure S5A,B) at 140, 168, and 196 d.p.i., which overlapped in time with the natural infection challenge of the main study. Whilst these calves were not tested for MERS-CoV RNA, this finding indicates that these calves were infected when the challenge study took place in the pen 200 m away. We strongly exclude the possibility of mechanical transfer of the virus due to the strict adherence of all staff to infection control measures, monitored by surveillance cameras, virus transmission and circulation to these calves through air could be possible. Of note, the two ELISAs were significantly correlated (Figure S5C).

## Discussion

In this study, we demonstrated that boosting humoral immune responses in MERS-CoV seropositive camels requires a single intramuscular dose of ChAdOx1 MERS, whereas at least two doses of vaccine were required to induce Abs in young seronegative camels (<1 year). Interestingly, older seronegative calves (1–2 years) responded to a single dose of the vaccine and elicited systemic and local nAbs. As the history of MERS-CoV exposure in these camels is not known, it is possible that older camels found here to be seronegative may have had some natural exposure to MERS-CoV but their Abs had then declined prior to their inclusion in the study as reported previously^25,27^. Nonetheless, it is also possible that these seronegative calves were truly immune naive to MERS-CoV and that greater vaccine responsiveness occurs in older animals with a more mature immune system. It is very unlikely that maternal Abs play any part in the lack of vaccine responsiveness in younger calves because ChAdOx1 MERS is a viral vectored vaccine that does not express MERS S protein on the virion surface and so will not be affected by pre-existing Abs at the time of vaccination.

Vaccination resulted in a statistically significant reduction in virus shedding after co-housing with infectious camels in both seropositive and seronegative camels. Whereas virus shedding was minimal from seropositive vaccinated camels, further optimisation is required to enhance protection in seronegative camels including the number of doses and route of immunisation. Indeed, assessing the ability of an MVA-vectored MERS-CoV vaccine to protect camels against contained MERS-CoV challenge required administering multiple doses by both intramuscular and intranasal routes to significantly reduce virus shedding^36^. Therefore, protective effects of vaccine administered by either the intranasal or intramuscular route should be assessed. Of note, we show here that in older seronegative camels, nAbs in nasal secretions were induced after a single intramuscular vaccination of ChAdOx1 MERS, but cannot rule out the possibility that these camels had previously been exposed to MERS-CoV despite proving seronegative on entry to the study, and the effects of intramuscular boosting after initial mucosal priming may be different from intramuscular priming of truly MERS-naive camels. The mechanism of vaccine-mediated or naturally acquired protection against MERS-CoV in camels is not yet known and further work will be required to study T cell responses as well as the possible contribution of immunity to antigens other than the S protein. However, there is lack of commercially available dromedary antibodies against immune cell markers, making T cell immunity in camels difficult to assess appropriately.

The ‘natural challenge’ model employed here replicates closely the conditions found in camel herds, and results in high levels of virus exposure over many days. Infectious camels were not removed from the pen after co-housing to mimic natural conditions. Even under these stringent conditions one camel in group +V demonstrated complete absence of virus shedding, with two others (out of a total of 5) in the same group shedding extremely low levels of virus, indicating that high levels of protective immunity can be achieved. Importantly, this group had high level of nAbs that plateaued for a year, unlike the +C group that had decreasing nAb titres overtime. These results suggest that vaccinating seropositive camels with S-based vaccine could boost their pre-existing MERS-specific immunity and minimise viral shedding. Nonetheless, following single dose vaccination, virus shedding was reduced in both seropositive and seronegative camels, which would reduce the likelihood of transmission to other camels or humans.

Previous work as well as ours have shown excellent correlation between pseudotyped and live virus neutralization assays^37^. Thus, it is expected that most nAbs detected by MERSpp neutralization assay bind to RBD or close to the binding site in a manner that blocks binding to the DPP4 cellular receptor, although the binding sites of nAbs detected in the pseudotyped virus neutralization assay have not been determined *per se*. Nonetheless, non-neutralising S-specific Abs could also interfere with MERS-CoV entry and fusion in a similar manner to stem-specific anti-influenza hemagglutinin Abs^38^, but this has not yet been assessed for MERS-CoV. Similarly, S1-based ELISA have shown excellent correlation with live virus neutralization assay although both nAbs as well as non-neutralising binding Abs could be detected by ELISA^39,40^. In the current study, we found that induction of S1-binding non-neutralising Abs alone is insufficient to reduce viral shedding and only the presence of nAbs can minimise virus excretion. Nonetheless, previous studies suggest that only presence of high levels on nAb titres may provide sterilising immunity in dromedaries as even seropositive camels could be re-infected as nAbs wane over time^25–28^. Our study also clearly confirms that MERS-CoV re-infection occur in dromedaries.

Of note, nasal discharge following infection was only observed in experimental setting and not in the market from which infectious camels were purchased, so may not be used as a reliable indicator of infection, supported also by previous studies^36,41^. Virus shedding continued for up to 14 days, indicating an average time for the course of MERS infection in pen-housed camels. Notably, although seronegative younger calves responded with high level of Abs after prime-boost, as mentioned above, they did not show significant different in nasal discharge nor in virus titre, as compared to the prime-only seronegative younger calves (in group −V). This indicate that inducing protective immunogenicity in seronegative calves needs stringent and optimal vaccination regimens.

Long-range airborne transmission of virus (to camels housed separately and managed under strict infection control measures) was also demonstrated, as was the case in the Korean hospital outbreak^42^. Accordingly, prevention of transmission between camels whenever large groups of animals are present, such as a market, large herd, or festival, cannot be prevented by hygiene measures only; and vaccination of camels should be introduced to minimise transmission between camels, and to humans. This study also presents a significant correlation between in-house ELISA and commercially available ELISA kits although low level of antibodies in some samples were detected only by in-house ELISA, previous reports showed that commercial ELISA kits may have low sensitivity^40^.

## Methods

### Pens, camels, personnel, and infection control

A camel research farm was set up 100 km from Riyadh city, remote from urban areas, with double fences and a secured gate. Inside this farm, three metal pens were set up with 30 m^2^ in size. Each pen is 150 cm height and has an infection control entry and exit points 10 m away from each other and from the pen. Two surveillance cameras were installed for each pen. Food and water troughs were placed inside each pen, where they can be filled from outside without entering the pens, supplementary file shows the farm layout. Nineteen seronegative camel calves (negative by ELISA), were sourced and purchased from Jouf province, north of Saudi Arabia, and transported for more than a 1000 km, using disinfected lorries, to the research farm. Additional ten seropositive and MERS-CoV RT-PCR negative camel calves were purchased from a camel market in Riyadh. Ten seronegative and seropositive calves were used in the main study whereas the remaining nine seronegative calves were used in a small follow up study to determine the age effect. Additional ten actively MERS-CoV shedding/infected camels (positive by RT-PCR; Ct value below 30) were purchased from local markets and co-housed with the experimental calves in one pen to serve as a natural infection model of challenge. Overall, a separate pen was used for each of the two studies and the third pen was only briefly used to gather infectious viral shedding camels one day before the challenge. All camels were labelled with paint as well as digital microchips; these two labels were used for the samples to ensure accuracy.

Pen keepers, veterinarians, and drivers were trained for infection control practices including fit-test for N95 masks. Each worker used their own specific N95 mask, overall white gown, goggles, head cover, shoe cover, all of which disposable and used once only. The team adhered to utilising the entry and exit point of each pen to ensure infection control; each pen, donning, and doffing area has biohazard waste container, sharp biohazard containers, 70% Ethanol spray and virucidal ANIOSpray (Laboratoires Anios, France) used by the workers. All staff involved in the study were also screened and confirmed MERS-CoV negative by ELISA and PCR prior to, during, and at the end of the study. Biowaste was collected daily and sent for incineration by the Saudi Gulf Environmental Protection Company (SEPCO). Clean sand from a nearby dunes area was used as pen floors, and new sand were added every 3 months. Pesticide was sprayed over the pen floors before starting the study or when new sand is added. The research farm and pens were monitored by surveillance camera to ensure strict adherence to infection control measures and to ensure camels are not exposed to any stray animals (e.g. fox, dogs). Some photographs of this study are included in the supplementary file.

### Vaccine, vaccination and sample collection

The ChAdOx1 MERS vaccine candidate and ChAdOx1-eGFP vector control were produced by the Jenner Institute at the University of Oxford, UK as previously described^30^. The vaccine and the vector control were transferred in a cold-chain delivery to KAIMRC, Riyadh, Saudi Arabia. They were thawed on ice once for dose preparation and a second time for a few minutes just before injections. Single doses of 1 × 10^9^ Infectious Unit (IU) of either the vaccine or the vector control in 1 ml PBS per camel were given intramuscularly into the thigh muscle. In the camel control groups of our studies (groups −C, +C, and AG01), camels were either immunised with ChAdOx1-eGFP (vector control) or PBS (placebo control). Blood samples were collected at different d.p.i. and transferred to the lab in portable refrigerators. For serum, 10 ml blood from each camel was collected at each time point. Blood was left to clot for 1 hr at 4 °C then spun at 15,000 rpm for 5 min and serum was collected, aliquoted and stored at −80 °C until testing. Nasal swabs from camels were collected in virus transport media (VTM) and stored at −80 °C until testing.

### ELISA

Serum samples were screened by ELISA using camel MERS-CoV EUROIMMUN ELISA kit (EUROIMMUN, Germany) according to the manufacturer’s instructions. Readouts were reported as the ratio of sample optometric density (OD) over the OD of an internal calibrator. Ratios of ≥1.1 and 0.8–1.1 were assigned as positive and borderline, respectively. Positive and negative controls provided with the kit were always included in each run. Endpoint titres of serum Abs were also determined using an in-house indirect ELISA as previously described^43,44^. Briefly, 96-well Nunc ELISA plates (Thermo Scientific, USA) coated with 50 μl/well of 1 μg/ml of recombinant S1 protein (IVI, Seoul, Korea) overnight, were washed and blocked for 1 h. Then, 50 μl/well from each serum sample was added in duplicates in a 3-fold serial dilution starting from a 1:100. After 1 h of incubation, alkaline phosphatase-conjugated rabbit anti-llama IgG (Agrisera, USA) were added at 1:3000 dilution. Finally, p-nitrophenyl phosphate substrate (pNPP) (Sigma Aldrich, Germany) was used for colour development and the OD was measured at 405 nm. The endpoint titre for each tested serum was determined as the reciprocal value of the serum dilution with OD value converging with the cut-off determined as the average OD of a seronegative camel serum plus 3 SD as described previously. The fold increase in Ab titres was calculated by dividing titres from each time point over the titre of the same camel on day 0, pre-vaccination titre (0 d.p.i.).

### MERS pseudotyped viral particles (MERSpp) neutralisation assay

MERS pseudotyped viral particles (MERSpp) were produced and titrated using Huh7.5 cells as described previously^45^. Heat-inactivated camel serum samples and nasal swab in VTM were prepared in a 3-fold serial dilution starting from 1:20, and tested for nAbs in duplicate as described previously^45^. A standard concentration of MERSpp (equivalent to 200,000 RLU) and Huh7.5 cells (10,000 cells) were added to each well. Cells only and cells with MERSpp only (both without serum) were included in quadruplicate as controls to determine 100% and 0% neutralisation activity, respectively. Following 48 h incubation, cells were lysed and the assay was developed using Bright-Glo™ Luciferase Assay System (Promega, USA) and luciferase activity was measured using a luminometer. IC_50_ neutralisation titres (in Log10) were calculated for each serum sample using GraphPad Prism. Experiments were performed twice independently. The background signal values of non-specific neutralisiation in sera from seronegative camels, pre-vaccination (samples of group –C at 0 d.p.i), were subtracted from all values of all samples (group –V, +C, and +V as well as from –C).

### Virus neutralisation assay (VNA)

A virus neutralisation assay was performed as previously described and the induction of virus nAbs was confirmed according to previously published protocols^30,46^. Briefly, heat-inactivated camel serum samples were tested for their capacity to neutralise MERS-CoV (EMC/2012 isolate) infections *in vitro* with nAb titre determined as the reciprocal of the highest dilution that completely protected Huh-7 cells in all tested wells (MN_100_).

### Nasal discharge scoring

As an arbitrary measure to evaluate camel MERS infections, nasal discharges from camels in all groups were observed daily and were given a score for the discharge abundance and severity; a score of 0 (for normal and healthy looking camel), 0.25 (if a camel had recovered from nasal discharge but with little remaining dry discharge), 1 (mild nasal secretion), 2 (moderate nasal secretion), and 3 (severe nasal secretion). Two vets were assigned to document the scores, also utilising camel nasal photographs taken in the first three days post challenge.

### RT-qPCR for viral RNA

The viral RNA was extracted from nasal swabs using MagNA Pure 96 DNA and Viral NA Small Volume Kit (Roche Diagnostics, USA). Extracted RNA samples were tested using one step RT-PCR targeting MERS-CoV UpE and ORF1a genes as previously described^47^ on LightCycler 480II (Roche Diagnostics, USA). Samples were considered positive only if both UpE and ORF1a amplicons were detected with Ct values ≤37. The Ct values of UpE gene were reported here.

### Viral titration by RT-qPCR

For the genome equivalent (GE) to the titre of 50% tissue culture infectious dose per ml (MERS-CoV GE (TCID_50_/ml)), MERS-CoV/Hu/Taif/SA/2015 isolate was cultured and titrated in Vero cells; and 1.5 × 10^7^ TCID_50_/ml was used to extract viral RNA. The viral RNA was serially diluted at 1:10 dilution factor then each dilution was used to synthesise cDNA. Camel nasal swabs in VTM were also used to extract total RNA and to synthesis cDNA. cDNA samples were used with ORF1a primers and probe (previously described^48^), to set a standard curve using TaqMan master mix and ABi 7500 Fast Real-Time PCR System (Applied Biosystems, USA). The cutoff of this assay was determined based on the last dilution before the plateau of the standard curve of the cultured viral RNA. Negative nasal swab samples from healthy humans and healthy camels were used to confirm this cutoff as shown in Figure S2B.

### Statistical analysis

Statistical analyses were performed to analyse the change in virus titres as well as nasal discharge scores over time post infection challenge. First, a mixed model for repeated measures design of the log-transformed MERS-CoV GE (TCID_50_/ml) values was employed to account for the covariance among repeated measures and assess the change in virus titres over time. Virus titres in all animals (subjects) were measured daily (equally spaced time points), the within-subject association among the repeated measures is modelled by assuming a first-order autoregressive correlation structure matrix. The same analysis model was also applied on the nasal discharge scores collected over time (from 1 to 14 days post challenge). Next, areas under the curve (AUC) of log-transformed MERS-CoV GE (TCID_50_/ml) values and nasal discharge scores collected from 1 to 14 d.p.c. for the two experimental groups (vaccinated and control) across the two conditions of seropositivity status (seropositive and seronegative) was reported. This analysis was conducted to display whether there are differences in virus titres nasal discharge scores between groups; it also includes Cohen’s *f*^2^ test, which is a standardised measure of effect size, ensuring the suitability of our study sample size for the statistical testing that we performed, see Supplementary statistic file. Statistical tests and analysis was conducted using SAS 9.4 (SAS Institute Inc., Cary, NC, USA) and GraphPad Prism software.

### Ethical approval

The study was approved by the Institutional Animal Care and Use Committee (IACUC) in King Abdullah International Medical Research Center (KAIMRC) in the Saudi ministry of National Guard – Health Affairs (MNG-HA). The animal study was conducted under supervision of the Saudi ministry of Environment, Water, and Agriculture (MEWA) and in accordance with the regulations of the law of ethics of research on living creatures, set and monitored by the Saudi National Committee of Bioethics (NCBE).

## Supplementary information

Supplementary file

## Data Availability

All data are available in this manuscript, no data in an archive.
