# Supplementary material for: Humoral Immunogenicity and Efficacy of a Single Dose of ChAdOx1 MERS Vaccine Candidate in Dromedary Camels
Source: Sci Rep. 2019 Nov 8;9:16292. doi: 10.1038/s41598-019-52730-4 (PMC6841732; doi:10.1038/s41598-019-52730-4)
Supplement: Supplementary file 1 — Supplementary file [file 41598_2019_52730_MOESM1_ESM.pdf]

Supplementary file

Article Title: Humoral Immunogenicity and Efficacy of a Single Dose of ChAdOx1 MERS Vaccine Candidate in Dromedary Camels

Authors: Naif Khalaf. Alharbi[1,2*], Ibrahim Qasim[3], Abdulrahman Almasoud[1], Haya A. Aljami[1], Mohamed W. Alenazi[1], Ali Alhafufi[3], Omar S. Aldibasi[4], Anwar M. Hashem[5,6], Samy Kasem[3,7], Raed Albrahim[3], Musaad Aldubaib[8], Ali Almansour[3], Nigel J. Temperton[9], Alexandra Kupke[10, 12], Stephan Becker[10,12], Ali Abu-obaidah[3], Ali Alkarar[3], In-Kyu Yoon[11], Esam Azhar[13,14], Teresa Lambe[15], Faisal Bayoumi[3], Ali Aldowerij[3], Osman H. Ibrahim[3], Sarah C. Gilbert[15], Hanan H. Balkhy[1,2,16]

Affiliations:

[1]Department of Infectious Disease Research, King Abdullah International Medical Research Center, Riyadh, Saudi Arabia

[2]King Saud bin Abdulaziz University for Health Sciences, Riyadh, Saudi Arabia

[3]Ministry of Environment, Water and Agriculture (MEWA), Riyadh, Saudi Arabia

[4]Department of Bioinformatics and Biostatistics, King Abdullah International Medical Research Center, Riyadh, Saudi Arabia

[5]Department of Medical Microbiology and Parasitology, Faculty of Medicine, King Abdulaziz University, Jeddah, Saudi Arabia

[6]Vaccines and Immunotherapy Unit, King Fahd Medical Research Center, King Abdulaziz University, Jeddah, Saudi Arabia

[7]Department of Virology, Faculty of Veterinary Medicine, Kafrelsheikh University, Kafrelsheikh, El Geish Street, 33516, Egypt

[8]College of Agriculture and Veterinary Medicine, Qassim University, Qassim, Saudi Arabia

[9]Viral Pseudotype Unit, Medway School of Pharmacy, University of Kent, Chatham, Kent, ME4 4TB, United Kingdom

[10]Institute of Virology, Philipps University of Marburg, Marburg, Germany

[11]International Vaccine Institute, Seoul, South Korea

[12]German Center for Infection Research, TTU Emerging Infections, Germany

[13]Department of Medical Laboratory Technology, Faculty of Applied Medical Sciences, King Abdulaziz University, Jeddah, Saudi Arabia

[14]Special Infectious Agents Unit, King Fahd Medical Research Center, King Abdulaziz University, Jeddah, Saudi Arabia

[15]The Jenner Institute, University of Oxford, Oxford OX3 7DQ, UK

[16]Department of Infection Prevention and Control, Ministry of National Guard - Health Affairs, Riyadh, Saudi Arabia

*To whom correspondence should be addressed: harbina2@nga.med.sa

I. Supplementary Figures

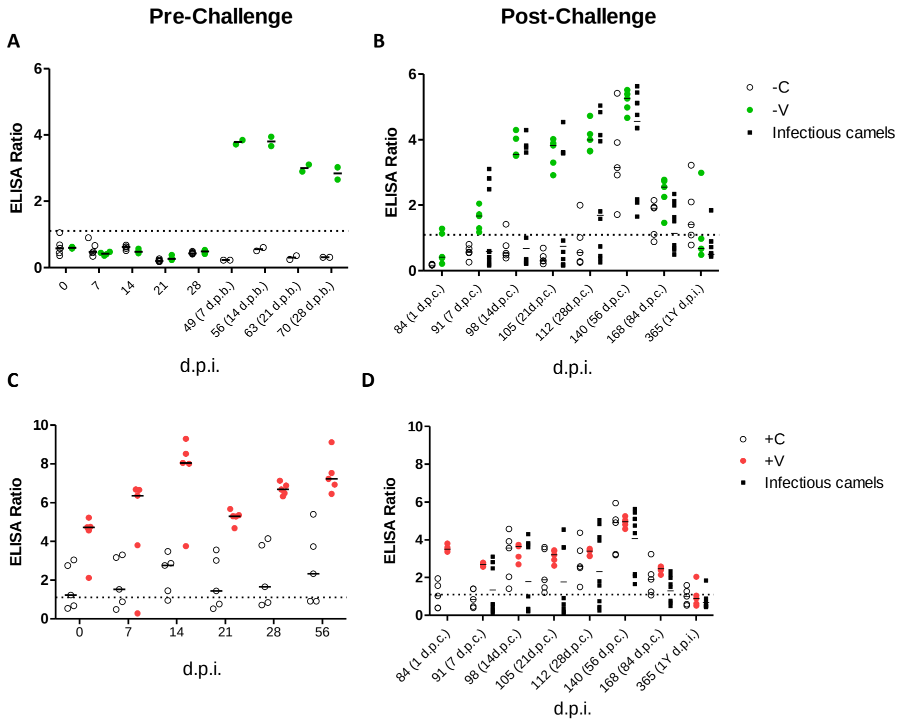

Figure S1: Antibody immune responses in dromedaries vaccinated with ChAdOx1 MERS assessed by Euroimmun ELISA kits.

Seronegative calves were either immunised with control injections (PBS or ChAdOx1 eGFP), group -C (open circles), or with ChAdOx1 MERS, group -V (green closed circles). Seropositive camels were either immunised with control injections (PBS or ChAdOx1 eGFP), group +C (open square), or with ChAdOx1 MERS, group +V (red closed square). Serum samples from different days post immunisation (d.p.i.) were evaluated in Euroimmun ELISA kits. Infectious camels used as a natural challenge models were also evaluated (black square). Data are plotted before the challenge (**A and C)** and after the challenge **(B and D)** with timepoints shown as d.p.i., days post boost (d.p.b.) and days post challenge (d.p.c.). Y: year; +: seropositive; -: seronegative; C: control group; V: vaccinated group.

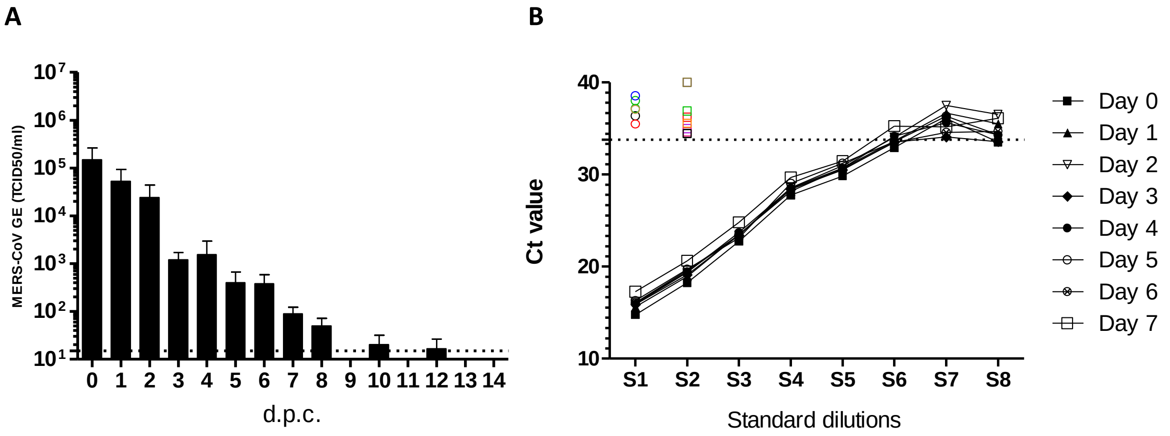

Figure S2: Genome equivalent ($TCID_{50}$/ml) titres in the MERS-CoV infectious camels.

A: Virus titres in MERS-CoV infectious camels used as a natural infection model were evaluated for 14 days post challenge (d.p.c.) using standard curve based RT-qPCR assay to report MERS-CoV GE ($TCID_{50}$/ml). Dotted line represents the detection limit of the assay, which is determined in graph **B**. **B:** Viral RNA from a sample with a known $TCID_{50}$/ml titre was utilised to set up the standard curve in RT-qPCR to evaluate the MERS-CoV GE ($TCID_{50}$/ml) titres. The detection limit of the assay was considered as the value of the last dilution before the plateau curve. Coloured circle and square symbols indicate values of negative samples from healthy camel and human, respectively.

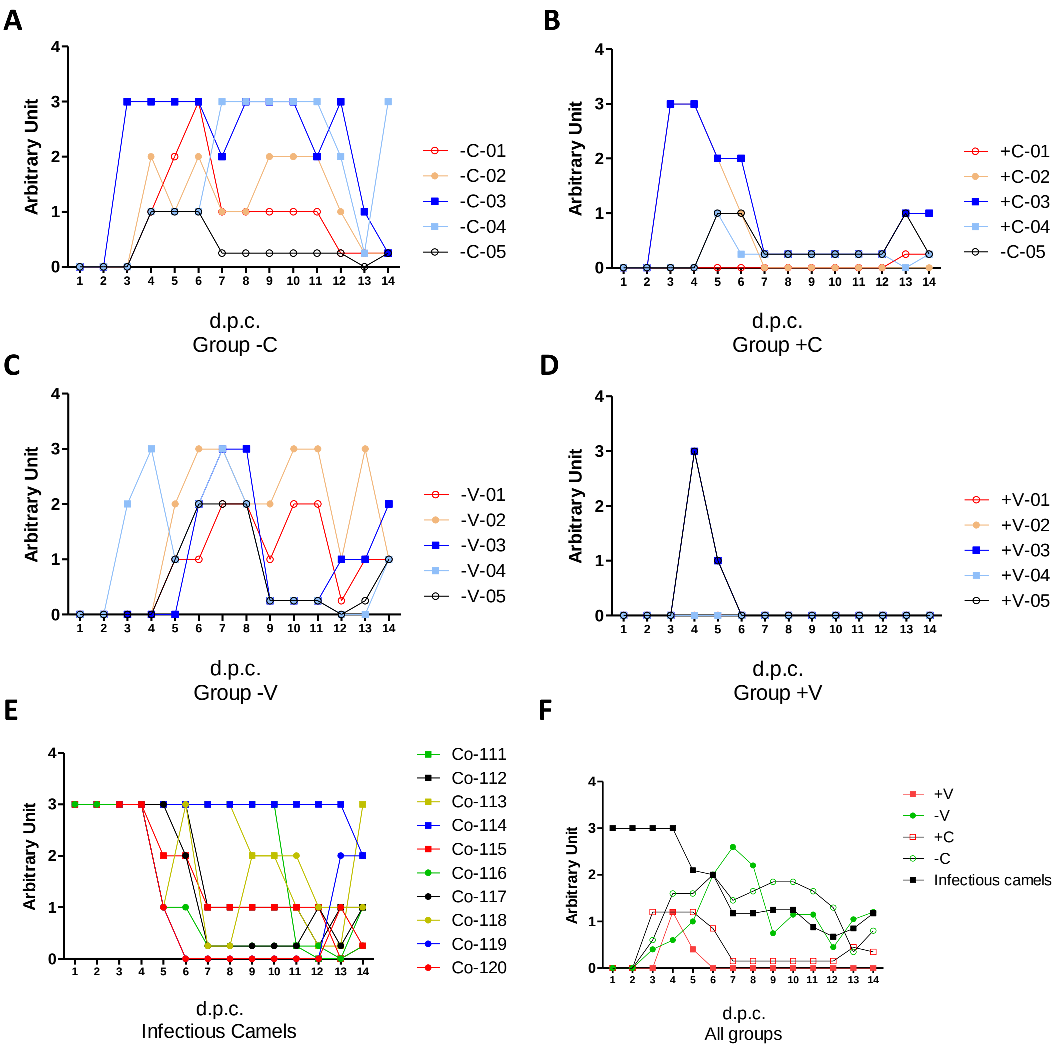

Figure S3: Severity and abundance of nasal discharge post natural infection challenge.

A-D: Scores of nasal discharge are reported for the twenty camels in all experimental groups of -C, -V, +C, and +V for 14 d.p.c. **E:** scores for the ten naturally infectious camels (used as a challenge model). **F:** average score per group. The scores were recorded as arbitrary units of 0 (normal), 0.25 (recovered), 1 (mild), 2 (moderate), or 3 (severe) depending on the nasal discharge abundance. Statistical analysis on areas under the curve of these scores is presented in Figure 3. +: seropositive; -: seronegative; C: control group; V: vaccinated group; Co-: co-housed camel.

A

d.p.c.	0	1	2	3	4	5	8	11	12	13	14	15	16	17	18	19	20	21	28	56
# infected camels	10	17	23	22	21	18	20	5	5	4	2	2	2	4	1	2	1	2	1	0

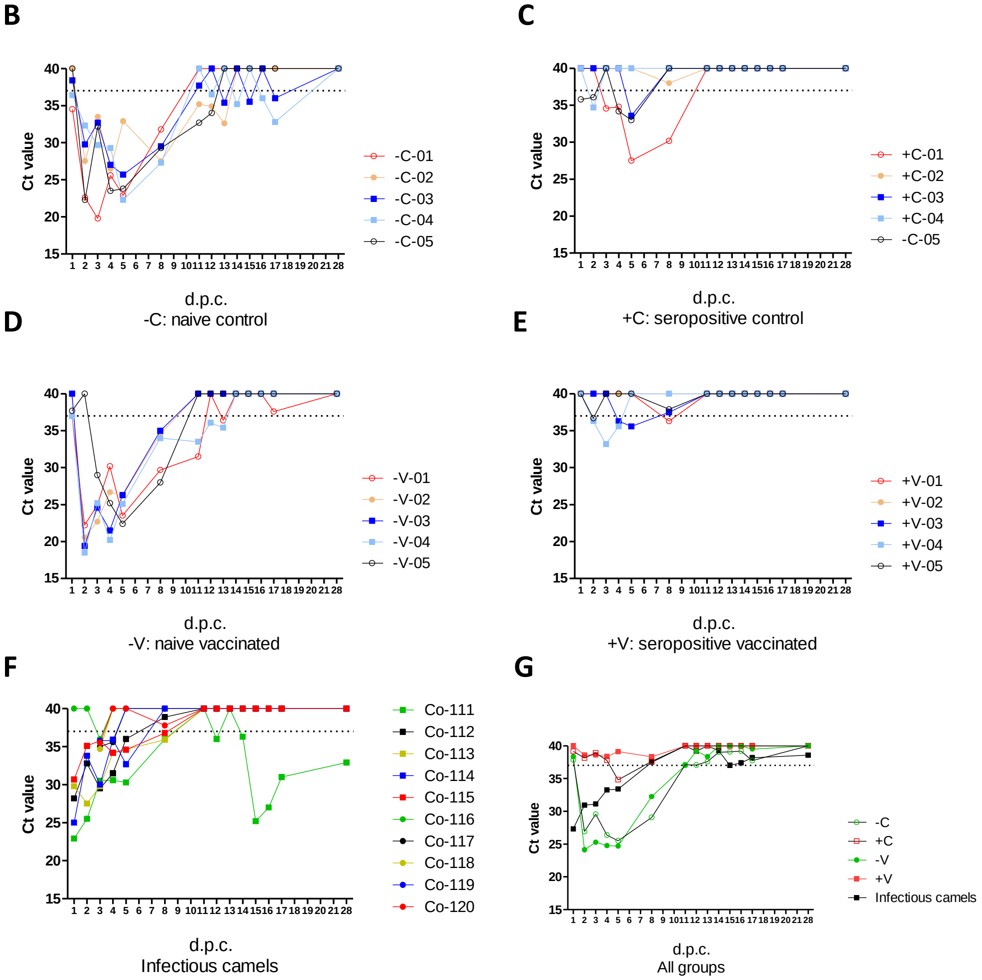

Figure S4: Efficacy of ChAdOx1 MERS in dromedaries based on RT-qPCR assay.

A: Camels in all groups (total n=20) were co-housed with naturally infectious camels (n=10), and the number of infected camels increased at different days post challenge (d.p.c.) as shown in the heatmap table. Nasal swabs were assessed for the presence of MERS-CoV UpE gene by RT-qPCR for 21 d.p.c. and for 28 d.p.c. The Ct values are shown for individual camels in each group (Groups: -C **(B)**, +C **(C)**, -V **(D)**, or +V **(E)**), the infectious camels **(F),** and as mean Ct values for each group **(G)**. +: seropositive; -: seronegative (naive); C: control group; V: vaccinated group.

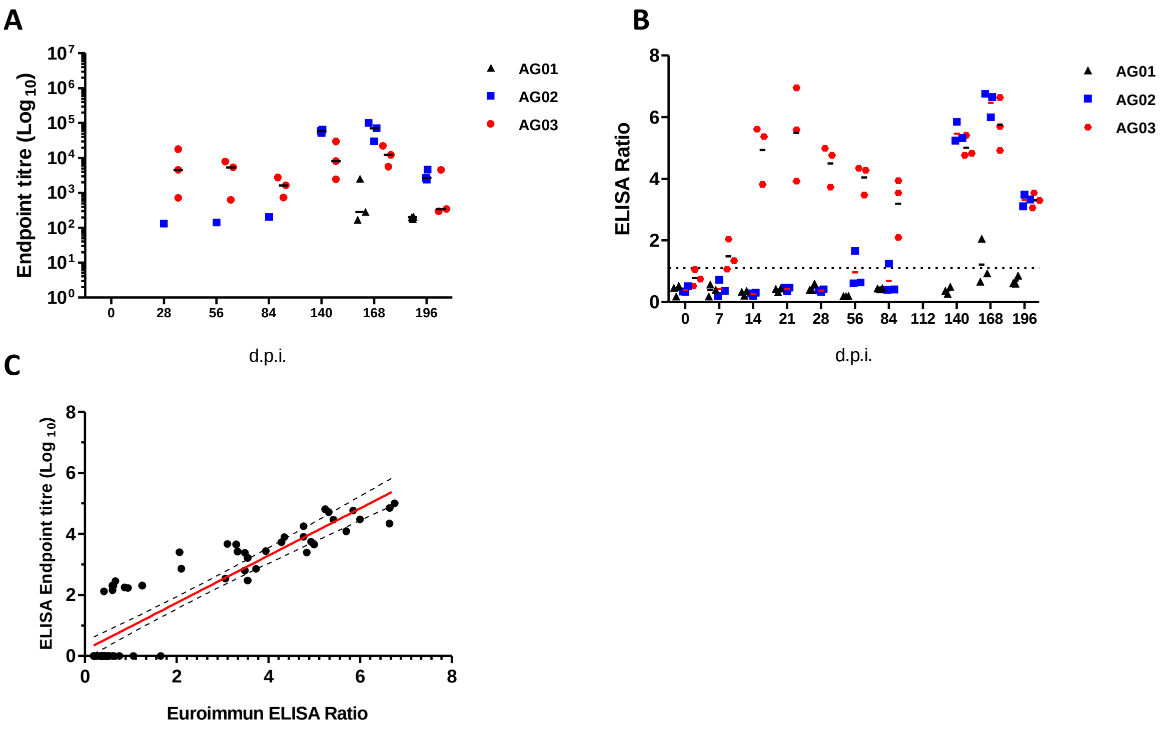

Figure S5: Antibody immune responses in different age of dromedary calves vaccinated with ChAdOx1 MERS assessed by Euroimmun ELISA kits.

Seronegative dromedary calves, below 1 year old, were immunised with PBS (n=2) or ChAdOx1-eGFP (n=1) in group AG01 (black triangles) or with ChAdOx1 MERS in group AG02 (n=3; blue squares). Older calves, below 2 years old, were immunised with ChAdOx1 MERS in group AG03 (n=3; red circles). Anti-spike antibody levels were assessed in endpoint titre ELISA **(A)** and Euroimmun ELISA kits **(B)**. Correlation between values in both assays is presented in (**C**). Linear regression between the antibody titres in the Endpoint ELISA and Euroimmun ELISA ratios showed a significant relationship with $R^2 = 0.82$ and $p < 0.0001$; and a significant correlation with Pearson $r = 0.90$ and $p < 0.0001$.

II. Supplementary Photographs

Photographs taken during the course of the study (*Reference to this article must be made when citing or sharing these photos*).

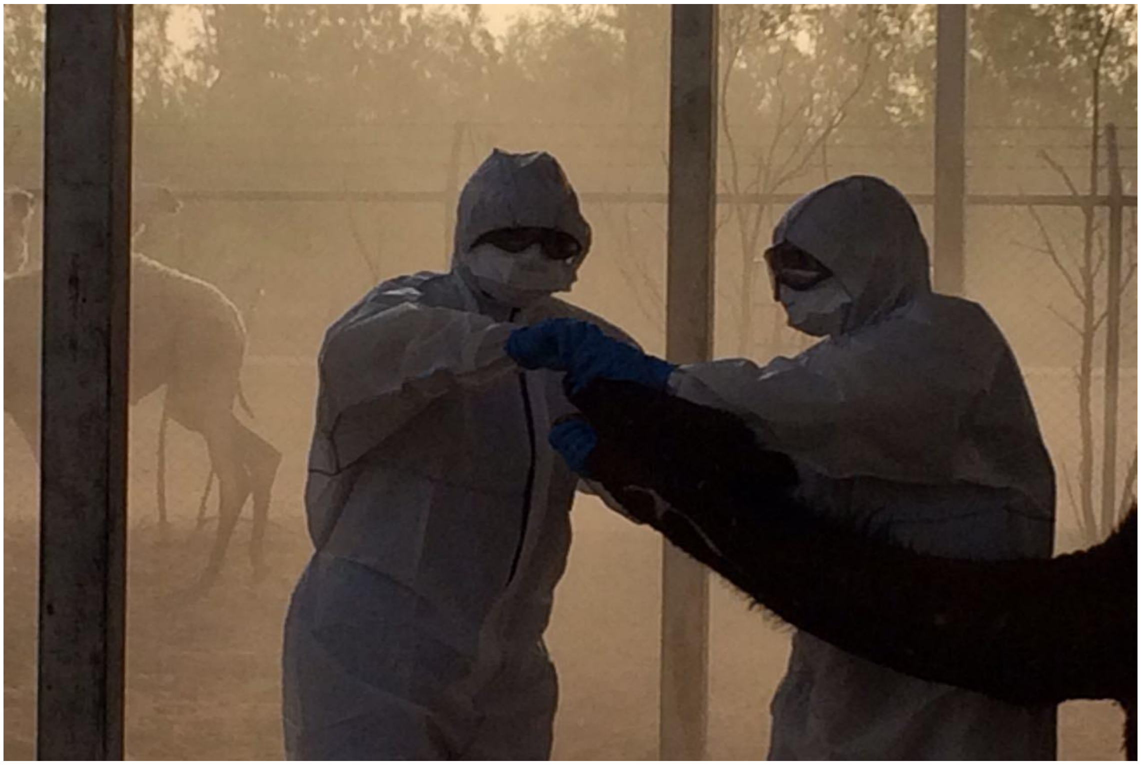

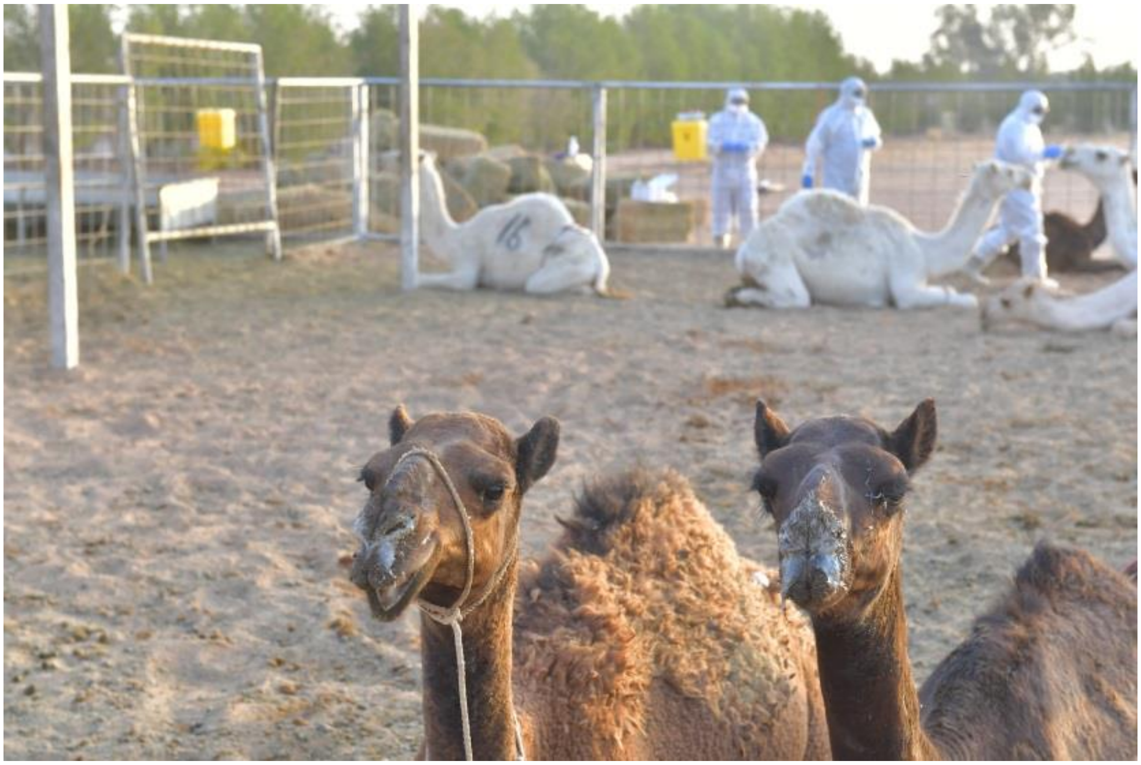

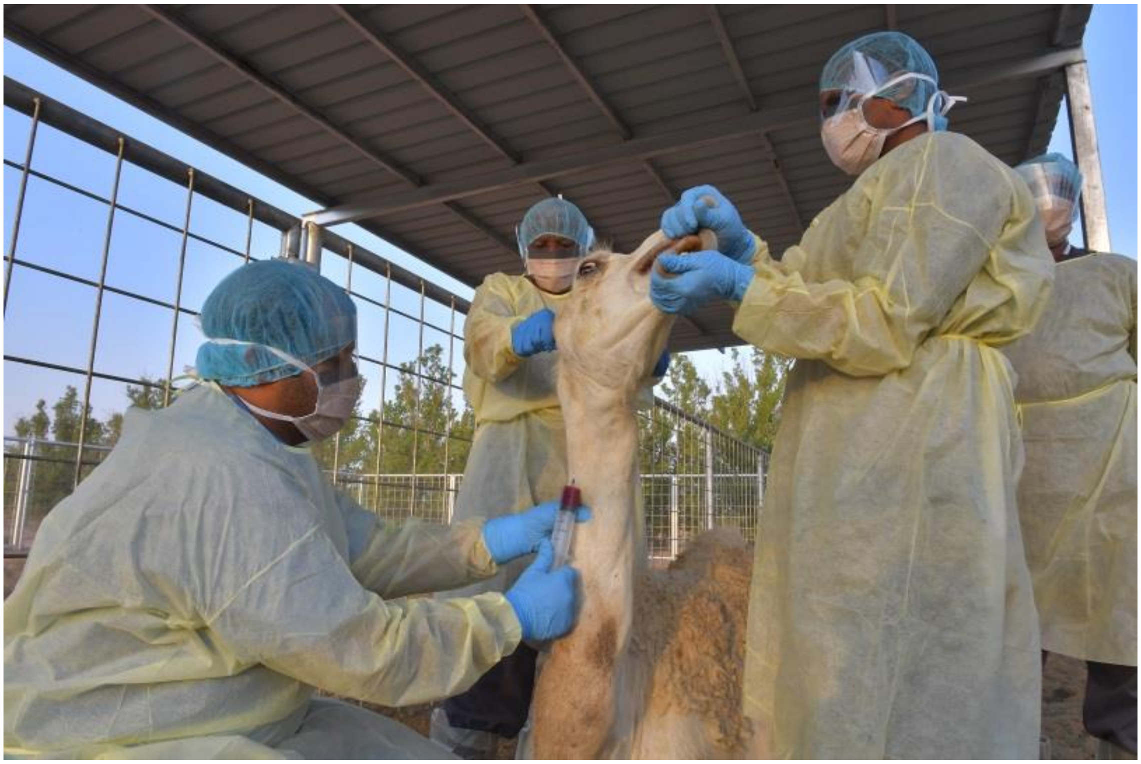

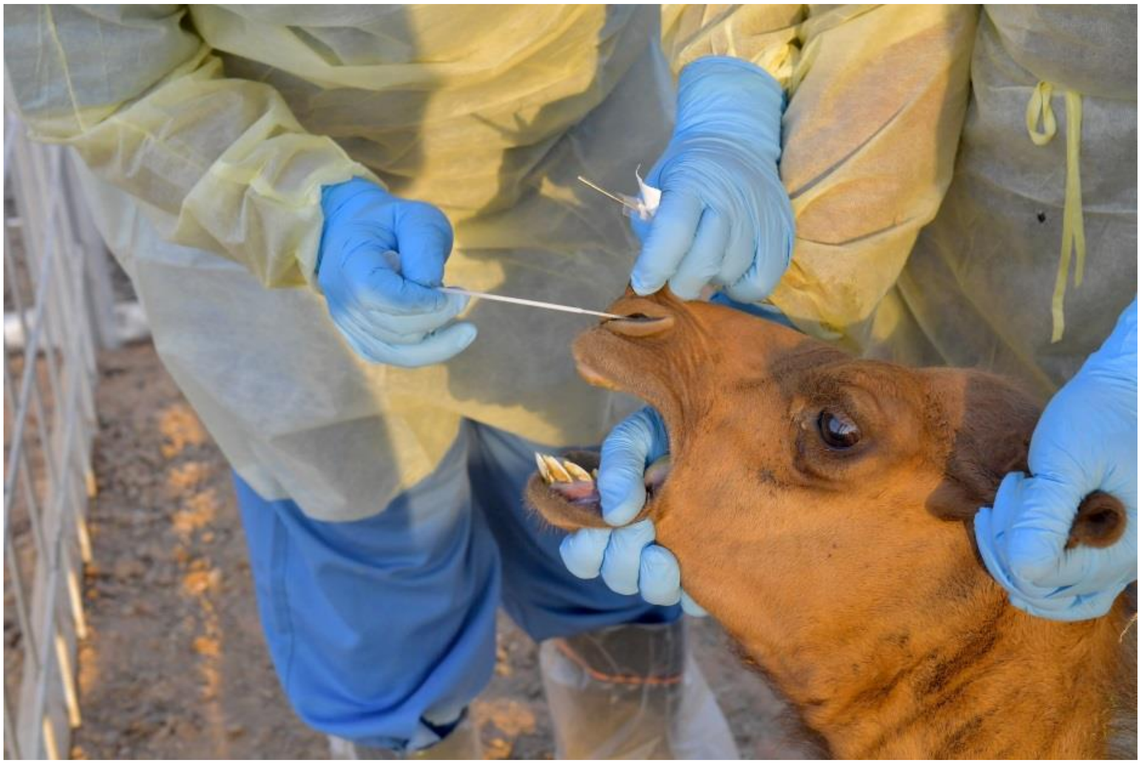

III. Supplementary diagram on the layout of the research farm

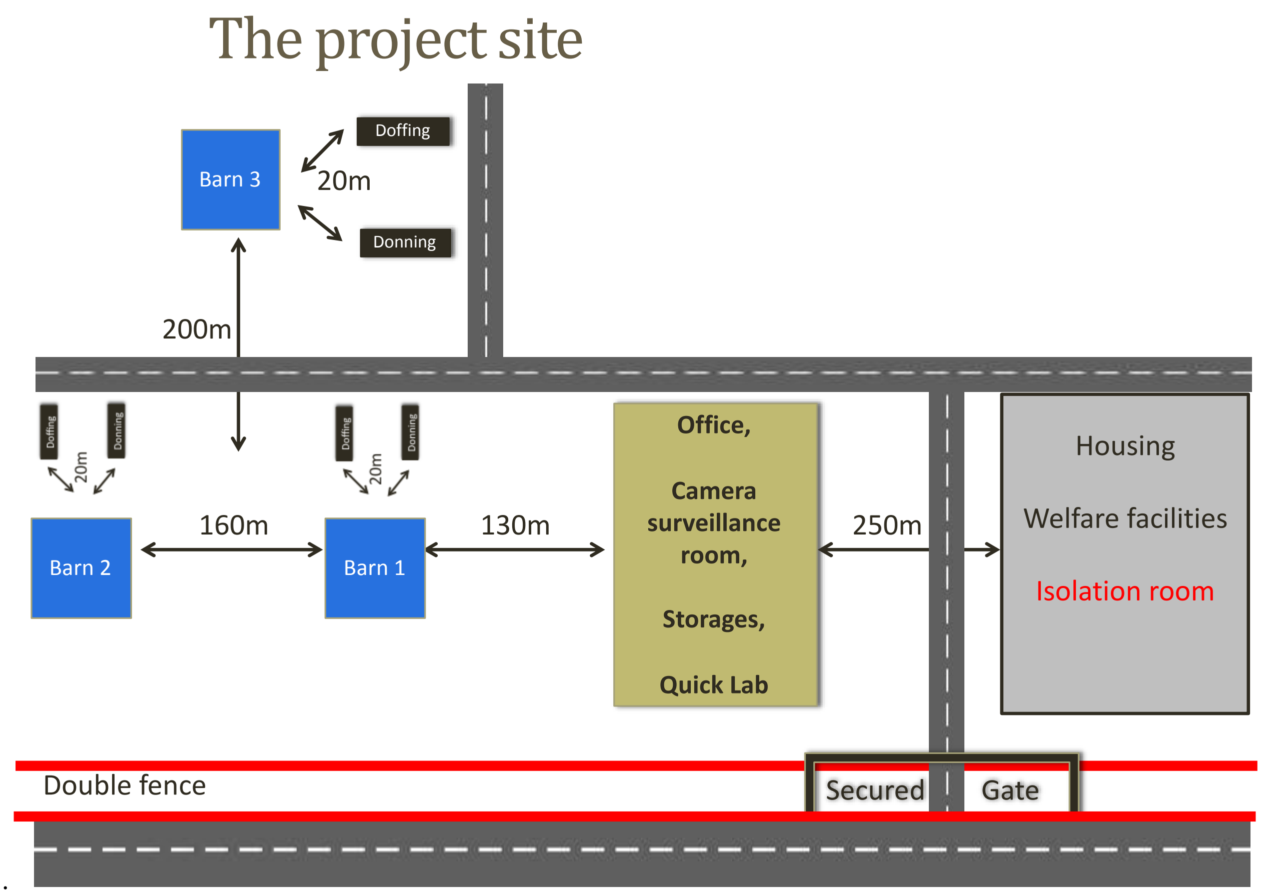

Distance is shown in metre (m). Each barn has donning and doffing dedicated areas

VI. Supplementary details on the statistical analysis

Note: Groups: -V (also called G01-N-V); -C (also called G02-N-C); +V (also called G03-S-V); +C (also called G04-S-C) where C: control, V: vaccinated, S: seropositive, N:naïve (seronegative).

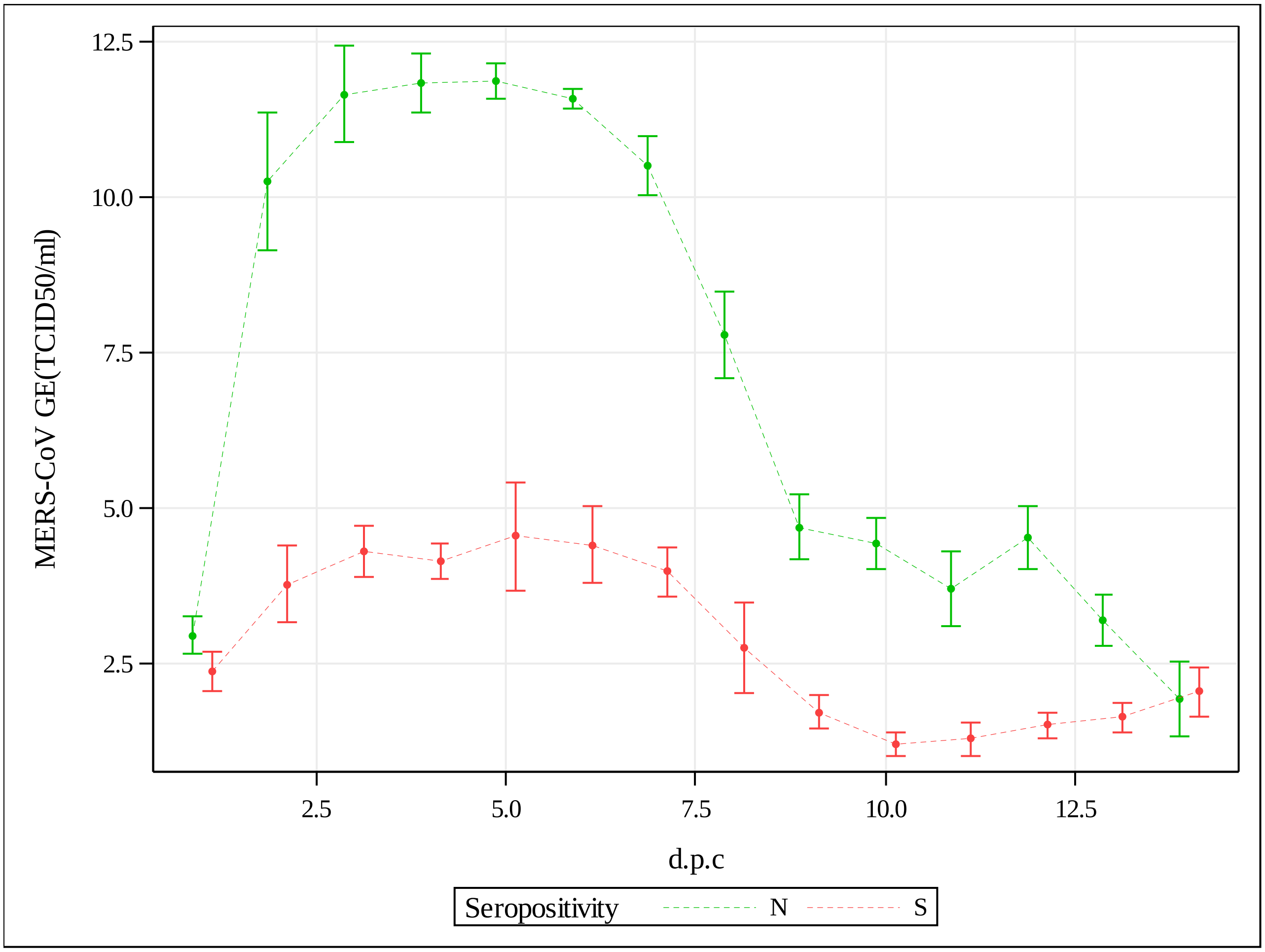

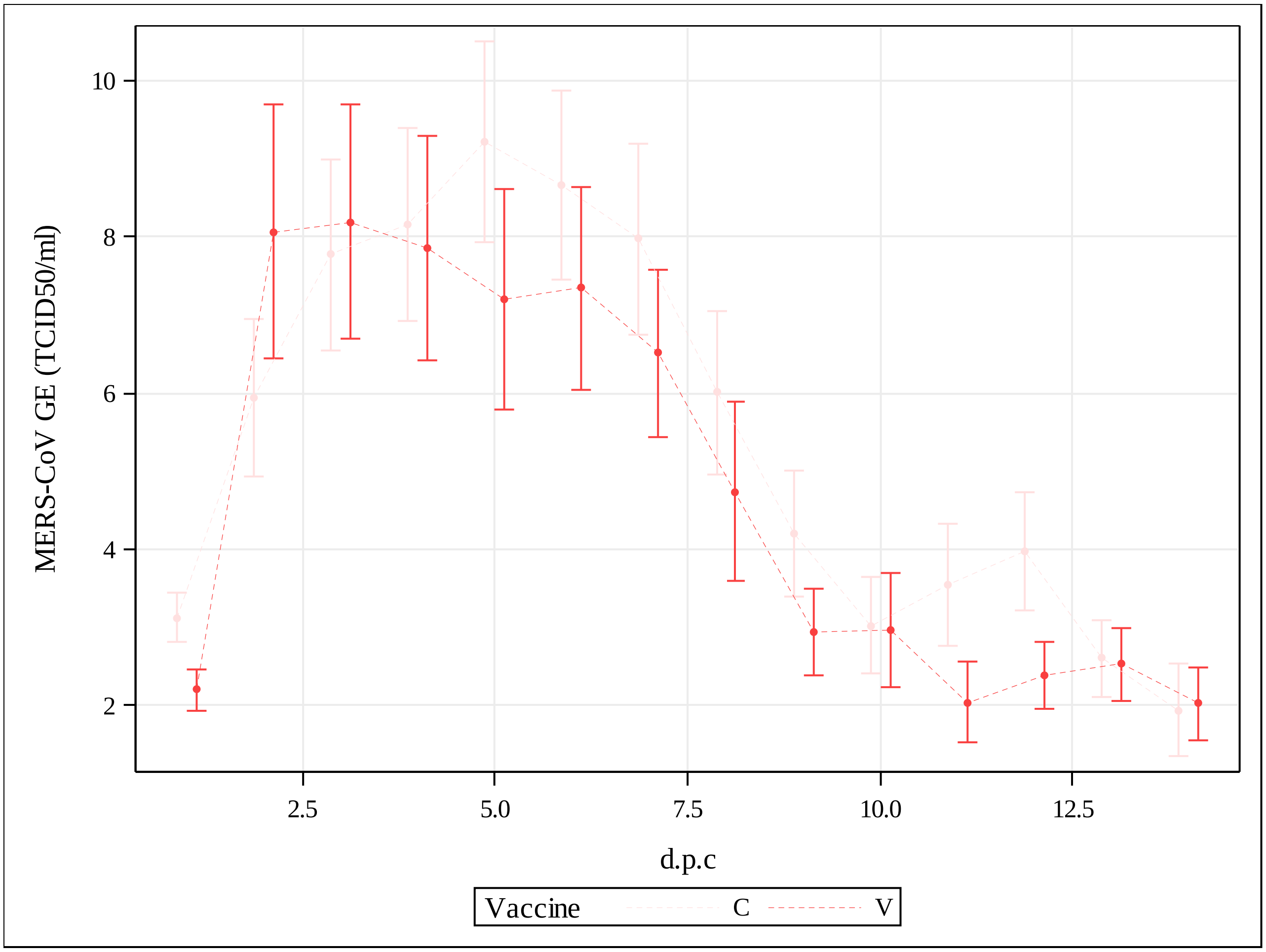
MERS-CoV GE (TCID50/ml)
10
8
6
4
2
2.5
5.0
7.5
10.0
12.5
d.p.c
Vaccine
C
V

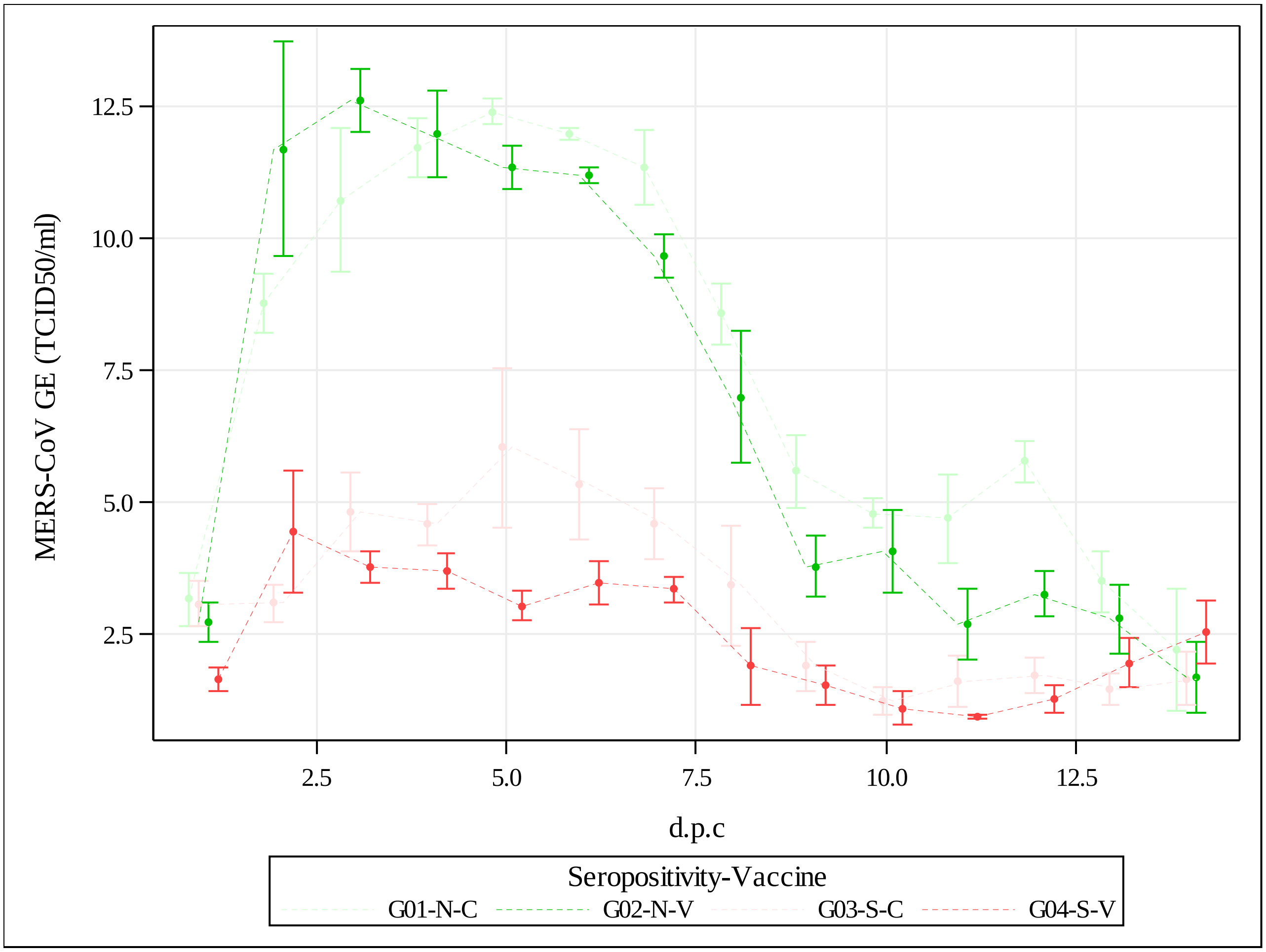
MERS-CoV GE (TCID50/ml)
12.5
10.0
7.5
5.0
2.5
2.5
5.0
7.5
10.0
12.5
d.p.c
Seropositivity-Vaccine
G01-N-C
G02-N-V
G03-S-C
G04-S-V

The Mixed Procedure

Model Information	
**Data Set**	WORK.DATA0
**Dependent Variable**	Virus_titre_post_challenge
**Covariance Structure**	Autoregressive
**Subject Effect**	CamelID
**Estimation Method**	REML
**Residual Variance Method**	Profile
**Fixed Effects SE Method**	Model-Based
**Degrees of Freedom Method**	Between-Within

Class Level Information		
**Class**	**Levels**	**Values**
**d_p_c_**	14	1 2 3 4 5 6 7 8 9 10 11 12 13 14
**CamelID**	20	11 12 13 14 15 21 22 23 24 25 31 32 33 34 35 41 42 43 44 45
**Seropositivity**	2	0 1
**Vaccine**	2	0 1

Dimensions	
**Covariance Parameters**	2
**Columns in X**	75
**Columns in Z**	0
**Subjects**	20
**Max Obs Per Subject**	14

Number of Observations	
**Number of Observations Read**	280
**Number of Observations Used**	258
**Number of Observations Not Used**	22

Iteration History			
**Iteration**	**Evaluations**	**-2 Res Log Like**	**Criterion**
**0**	1	888.78662828	
**1**	2	865.57005966	0.00000020
**2**	1	865.57001358	0.00000000

The Mixed Procedure

Convergence criteria met.

Estimated R Matrix for CamelID 11												
**Row**	**Col1**	**Col2**	**Col3**	**Col4**	**Col5**	**Col6**	**Col7**	**Col8**	**Col9**	**Col10**	**Col11**	**Col12**
**1**	2.4075	0.7709	0.2468	0.07904	0.02531	0.008104	0.002595	0.000831	0.000266	0.000085	0.000027	8.736E-6
**2**	0.7709	2.4075	0.7709	0.2468	0.07904	0.02531	0.008104	0.002595	0.000831	0.000266	0.000085	0.000027
**3**	0.2468	0.7709	2.4075	0.7709	0.2468	0.07904	0.02531	0.008104	0.002595	0.000831	0.000266	0.000085
**4**	0.07904	0.2468	0.7709	2.4075	0.7709	0.2468	0.07904	0.02531	0.008104	0.002595	0.000831	0.000266
**5**	0.02531	0.07904	0.2468	0.7709	2.4075	0.7709	0.2468	0.07904	0.02531	0.008104	0.002595	0.000831
**6**	0.008104	0.02531	0.07904	0.2468	0.7709	2.4075	0.7709	0.2468	0.07904	0.02531	0.008104	0.002595
**7**	0.002595	0.008104	0.02531	0.07904	0.2468	0.7709	2.4075	0.7709	0.2468	0.07904	0.02531	0.008104
**8**	0.000831	0.002595	0.008104	0.02531	0.07904	0.2468	0.7709	2.4075	0.7709	0.2468	0.07904	0.02531
**9**	0.000266	0.000831	0.002595	0.008104	0.02531	0.07904	0.2468	0.7709	2.4075	0.7709	0.2468	0.07904
**10**	0.000085	0.000266	0.000831	0.002595	0.008104	0.02531	0.07904	0.2468	0.7709	2.4075	0.7709	0.2468
**11**	0.000027	0.000085	0.000266	0.000831	0.002595	0.008104	0.02531	0.07904	0.2468	0.7709	2.4075	0.7709
**12**	8.736E-6	0.000027	0.000085	0.000266	0.000831	0.002595	0.008104	0.02531	0.07904	0.2468	0.7709	2.4075
**13**	2.797E-6	8.736E-6	0.000027	0.000085	0.000266	0.000831	0.002595	0.008104	0.02531	0.07904	0.2468	0.7709
**14**	8.957E-7	2.797E-6	8.736E-6	0.000027	0.000085	0.000266	0.000831	0.002595	0.008104	0.02531	0.07904	0.2468

Estimated R Matrix for CamelID 11		
**Row**	**Col13**	**Col14**
**1**	2.797E-6	8.957E-7
**2**	8.736E-6	2.797E-6
**3**	0.000027	8.736E-6
**4**	0.000085	0.000027
**5**	0.000266	0.000085
**6**	0.000831	0.000266
**7**	0.002595	0.000831
**8**	0.008104	0.002595
**9**	0.02531	0.008104
**10**	0.07904	0.02531
**11**	0.2468	0.07904
**12**	0.7709	0.2468
**13**	2.4075	0.7709
**14**	0.7709	2.4075

The Mixed Procedure

Estimated R Correlation Matrix for CamelID 11

Row	Col1	Col2	Col3	Col4	Col5	Col6	Col7	Col8	Col9	Col10	Col11	Col12
1	1.0000	0.3202	0.1025	0.03283	0.01051	0.003366	0.001078	0.000345	0.000111	0.000035	0.000011	3.629E-6
2	0.3202	1.0000	0.3202	0.1025	0.03283	0.01051	0.003366	0.001078	0.000345	0.000111	0.000035	0.000011
3	0.1025	0.3202	1.0000	0.3202	0.1025	0.03283	0.01051	0.003366	0.001078	0.000345	0.000111	0.000035
4	0.03283	0.1025	0.3202	1.0000	0.3202	0.1025	0.03283	0.01051	0.003366	0.001078	0.000345	0.000111
5	0.01051	0.03283	0.1025	0.3202	1.0000	0.3202	0.1025	0.03283	0.01051	0.003366	0.001078	0.000345
6	0.003366	0.01051	0.03283	0.1025	0.3202	1.0000	0.3202	0.1025	0.03283	0.01051	0.003366	0.001078
7	0.001078	0.003366	0.01051	0.03283	0.1025	0.3202	1.0000	0.3202	0.1025	0.03283	0.01051	0.003366
8	0.000345	0.001078	0.003366	0.01051	0.03283	0.1025	0.3202	1.0000	0.3202	0.1025	0.03283	0.01051
9	0.000111	0.000345	0.001078	0.003366	0.01051	0.03283	0.1025	0.3202	1.0000	0.3202	0.1025	0.03283
10	0.000035	0.000111	0.000345	0.001078	0.003366	0.01051	0.03283	0.1025	0.3202	1.0000	0.3202	0.1025
11	0.000011	0.000035	0.000111	0.000345	0.001078	0.003366	0.01051	0.03283	0.1025	0.3202	1.0000	0.3202
12	3.629E-6	0.000011	0.000035	0.000111	0.000345	0.001078	0.003366	0.01051	0.03283	0.1025	0.3202	1.0000
13	1.162E-6	3.629E-6	0.000011	0.000035	0.000111	0.000345	0.001078	0.003366	0.01051	0.03283	0.1025	0.3202
14	3.721E-7	1.162E-6	3.629E-6	0.000011	0.000035	0.000111	0.000345	0.001078	0.003366	0.01051	0.03283	0.1025

Estimated R Correlation Matrix for CamelID 11

Row	Col13	Col14
1	1.162E-6	3.721E-7
2	3.629E-6	1.162E-6
3	0.000011	3.629E-6
4	0.000035	0.000011
5	0.000111	0.000035
6	0.000345	0.000111
7	0.001078	0.000345
8	0.003366	0.001078
9	0.01051	0.003366
10	0.03283	0.01051
11	0.1025	0.03283
12	0.3202	0.1025
13	1.0000	0.3202
14	0.3202	1.0000

The Mixed Procedure

Covariance Parameter Estimates		
**Cov Parm**	**Subject**	**Estimate**
**AR(1)**	CamelID	0.3202
**Residual**		2.4075

Fit Statistics	
**-2 Res Log Likelihood**	865.6
**AIC (smaller is better)**	869.6
**AICC (smaller is better)**	869.6
**BIC (smaller is better)**	871.6

Null Model Likelihood Ratio Test		
**DF**	**Chi-Square**	**Pr > ChiSq**
1	23.22	<.0001

Solution for Fixed Effects								
**Effect**	**d_p_c_**	**Seropositivity**	**Vaccine**	**Estimate**	**Standard Error**	**DF**	**t Value**	**Pr > |t|**
**Intercept**				2.1258	0.7302	17	2.91	0.0097
**d_p_c_**	1			-0.2160	0.9457	199	-0.23	0.8195
**d_p_c_**	2			2.7107	0.9457	199	2.87	0.0046
**d_p_c_**	3			2.3857	0.9457	199	2.52	0.0124
**d_p_c_**	4			1.8748	0.9457	199	1.98	0.0488
**d_p_c_**	5			1.4098	0.9457	199	1.49	0.1376
**d_p_c_**	6			1.6282	0.9456	199	1.72	0.0867
**d_p_c_**	7			1.1233	0.9455	199	1.19	0.2363
**d_p_c_**	8			-0.1722	0.9725	199	-0.18	0.8597
**d_p_c_**	9			-0.9122	1.0268	199	-0.89	0.3754
**d_p_c_**	10			-1.1342	1.0120	199	-1.12	0.2638
**d_p_c_**	11			-1.5910	1.0177	199	-1.56	0.1196
**d_p_c_**	12			-1.4066	0.9349	199	-1.50	0.1340
**d_p_c_**	13			-0.4489	0.9779	199	-0.46	0.6467
**d_p_c_**	14			0	.	.	.	.
**Seropositivity**		0		-0.1757	0.7818	17	-0.22	0.8248

The Mixed Procedure

Solution for Fixed Effects								
**Effect**	**d_p_c_**	**Seropositivity**	**Vaccine**	**Estimate**	**Standard Error**	**DF**	**t Value**	**Pr > |t|**
**Seropositivity**		1		0	.	.	.	.
**Vaccine**			0	-0.1233	0.7757	17	-0.16	0.8755
**Vaccine**			1	0	.	.	.	.
**d_p_c_*Seropositivit**	1	0		0.7593	1.0454	199	0.73	0.4685
**d_p_c_*Seropositivit**	1	1		0	.	.	.	.
**d_p_c_*Seropositivit**	2	0		6.6415	1.0454	199	6.35	<.0001
**d_p_c_*Seropositivit**	2	1		0	.	.	.	.
**d_p_c_*Seropositivit**	3	0		7.5349	1.0453	199	7.21	<.0001
**d_p_c_*Seropositivit**	3	1		0	.	.	.	.
**d_p_c_*Seropositivit**	4	0		7.8667	1.0453	199	7.53	<.0001
**d_p_c_*Seropositivit**	4	1		0	.	.	.	.
**d_p_c_*Seropositivit**	5	0		7.5032	1.0453	199	7.18	<.0001
**d_p_c_*Seropositivit**	5	1		0	.	.	.	.
**d_p_c_*Seropositivit**	6	0		7.3501	1.0453	199	7.03	<.0001
**d_p_c_*Seropositivit**	6	1		0	.	.	.	.
**d_p_c_*Seropositivit**	7	0		6.7013	1.0452	199	6.41	<.0001
**d_p_c_*Seropositivit**	7	1		0	.	.	.	.
**d_p_c_*Seropositivit**	8	0		5.2587	1.0559	199	4.98	<.0001
**d_p_c_*Seropositivit**	8	1		0	.	.	.	.
**d_p_c_*Seropositivit**	9	0		3.0555	1.1063	199	2.76	0.0063
**d_p_c_*Seropositivit**	9	1		0	.	.	.	.
**d_p_c_*Seropositivit**	10	0		3.4176	1.0695	199	3.20	0.0016
**d_p_c_*Seropositivit**	10	1		0	.	.	.	.
**d_p_c_*Seropositivit**	11	0		2.6550	1.0945	199	2.43	0.0162
**d_p_c_*Seropositivit**	11	1		0	.	.	.	.
**d_p_c_*Seropositivit**	12	0		3.2084	1.0200	199	3.15	0.0019
**d_p_c_*Seropositivit**	12	1		0	.	.	.	.
**d_p_c_*Seropositivit**	13	0		1.5815	0.9793	199	1.61	0.1079
**d_p_c_*Seropositivit**	13	1		0	.	.	.	.
**d_p_c_*Seropositivit**	14	0		0	.	.	.	.
**d_p_c_*Seropositivit**	14	1		0	.	.	.	.
**d_p_c_*Vaccine**	1		0	1.0482	1.0407	199	1.01	0.3151
**d_p_c_*Vaccine**	1		1	0	.	.	.	.
**d_p_c_*Vaccine**	2		0	-2.0051	1.0407	199	-1.93	0.0554

The Mixed Procedure

Solution for Fixed Effects								
**Effect**	**d_p_c_**	**Seropositivity**	**Vaccine**	**Estimate**	**Standard Error**	**DF**	**t Value**	**Pr > |t|**
**d_p_c_*Vaccine**	2		1	0	.	.	.	.
**d_p_c_*Vaccine**	3		0	-0.3004	1.0407	199	-0.29	0.7732
**d_p_c_*Vaccine**	3		1	0	.	.	.	.
**d_p_c_*Vaccine**	4		0	0.4296	1.0407	199	0.41	0.6802
**d_p_c_*Vaccine**	4		1	0	.	.	.	.
**d_p_c_*Vaccine**	5		0	2.1447	1.0407	199	2.06	0.0406
**d_p_c_*Vaccine**	5		1	0	.	.	.	.
**d_p_c_*Vaccine**	6		0	1.4380	1.0407	199	1.38	0.1686
**d_p_c_*Vaccine**	6		1	0	.	.	.	.
**d_p_c_*Vaccine**	7		0	1.5824	1.0406	199	1.52	0.1299
**d_p_c_*Vaccine**	7		1	0	.	.	.	.
**d_p_c_*Vaccine**	8		0	1.6318	1.0514	199	1.55	0.1222
**d_p_c_*Vaccine**	8		1	0	.	.	.	.
**d_p_c_*Vaccine**	9		0	1.3215	1.0865	199	1.22	0.2253
**d_p_c_*Vaccine**	9		1	0	.	.	.	.
**d_p_c_*Vaccine**	10		0	0.5375	1.0649	199	0.50	0.6143
**d_p_c_*Vaccine**	10		1	0	.	.	.	.
**d_p_c_*Vaccine**	11		0	1.5011	1.0736	199	1.40	0.1636
**d_p_c_*Vaccine**	11		1	0	.	.	.	.
**d_p_c_*Vaccine**	12		0	1.6740	1.0126	199	1.65	0.0999
**d_p_c_*Vaccine**	12		1	0	.	.	.	.
**d_p_c_*Vaccine**	13		0	0.2197	0.9688	199	0.23	0.8208
**d_p_c_*Vaccine**	13		1	0	.	.	.	.
**d_p_c_*Vaccine**	14		0	0	.	.	.	.
**d_p_c_*Vaccine**	14		1	0	.	.	.	.

Type 3 Tests of Fixed Effects				
**Effect**	**Num DF**	**Den DF**	**F Value**	**Pr > F**
**d_p_c_**	13	199	35.21	<.0001
**Seropositivity**	1	17	284.28	<.0001
**Vaccine**	1	17	6.89	0.0177
**d_p_c_*Seropositivit**	13	199	11.44	<.0001
**d_p_c_*Vaccine**	13	199	2.36	0.0059

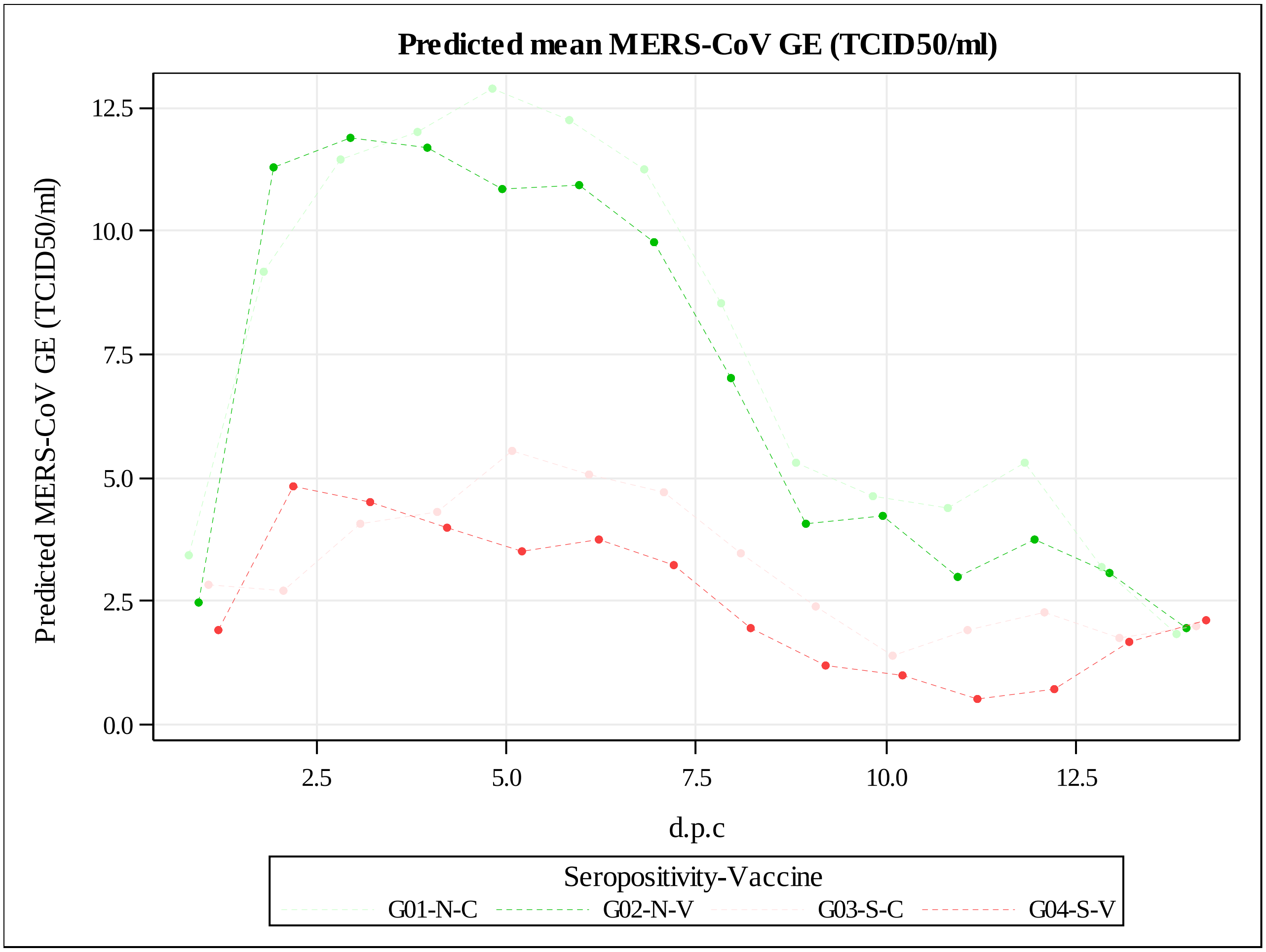
Predicted mean MERS-CoV GE (TCID50/ml)
Predicted MERS-CoV GE (TCID50/ml)
0.0
2.5
5.0
7.5
10.0
12.5
2.5
5.0
7.5
10.0
12.5
d.p.c
Seropositivity-Vaccine
G01-N-C
G02-N-V
G03-S-C
G04-S-V

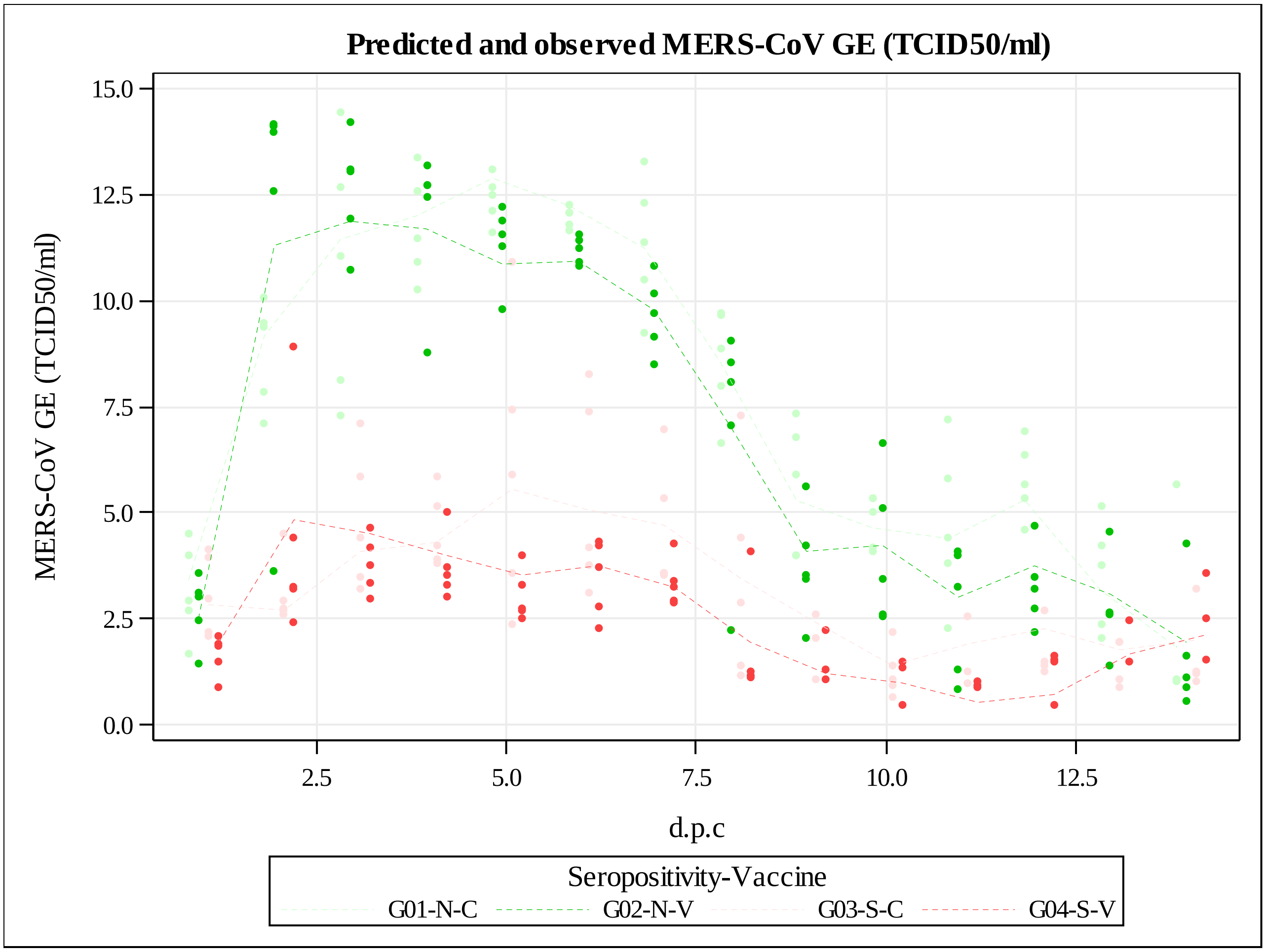
Predicted and observed MERS-CoV GE (TCID50/ml)
MERS-CoV GE (TCID50/ml)
0.0
2.5
5.0
7.5
10.0
12.5
15.0
2.5
5.0
7.5
10.0
12.5
d.p.c
Seropositivity-Vaccine
G01-N-C
G02-N-V
G03-S-C
G04-S-V

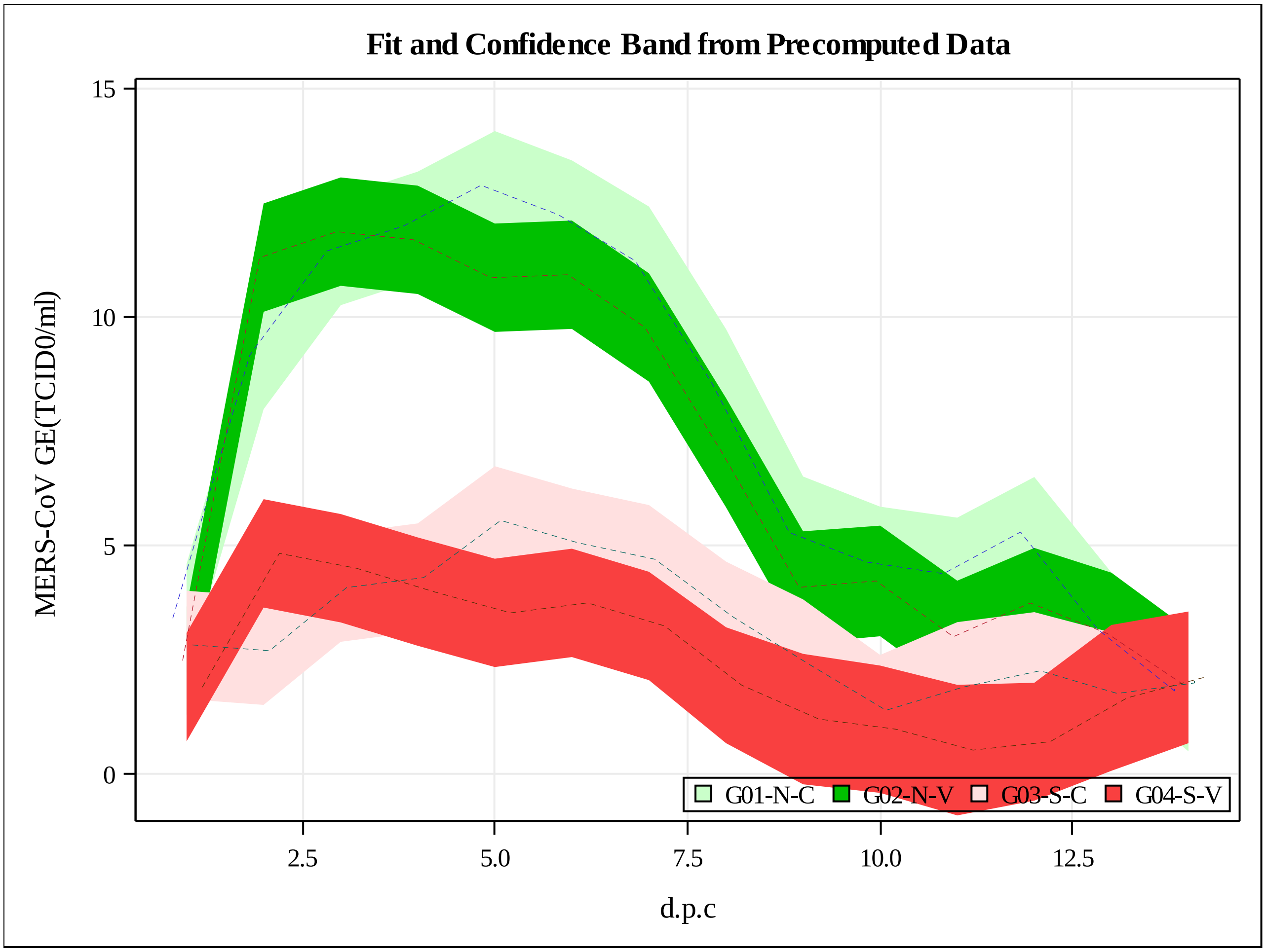

Fit and Confidence Band from Precomputed Data
MERS-CoV GE(TCID0/ml)
15
10
5
0
2.5
5.0
7.5
10.0
12.5
d.p.c
G01-N-C
G02-N-V
G03-S-C
G04-S-V

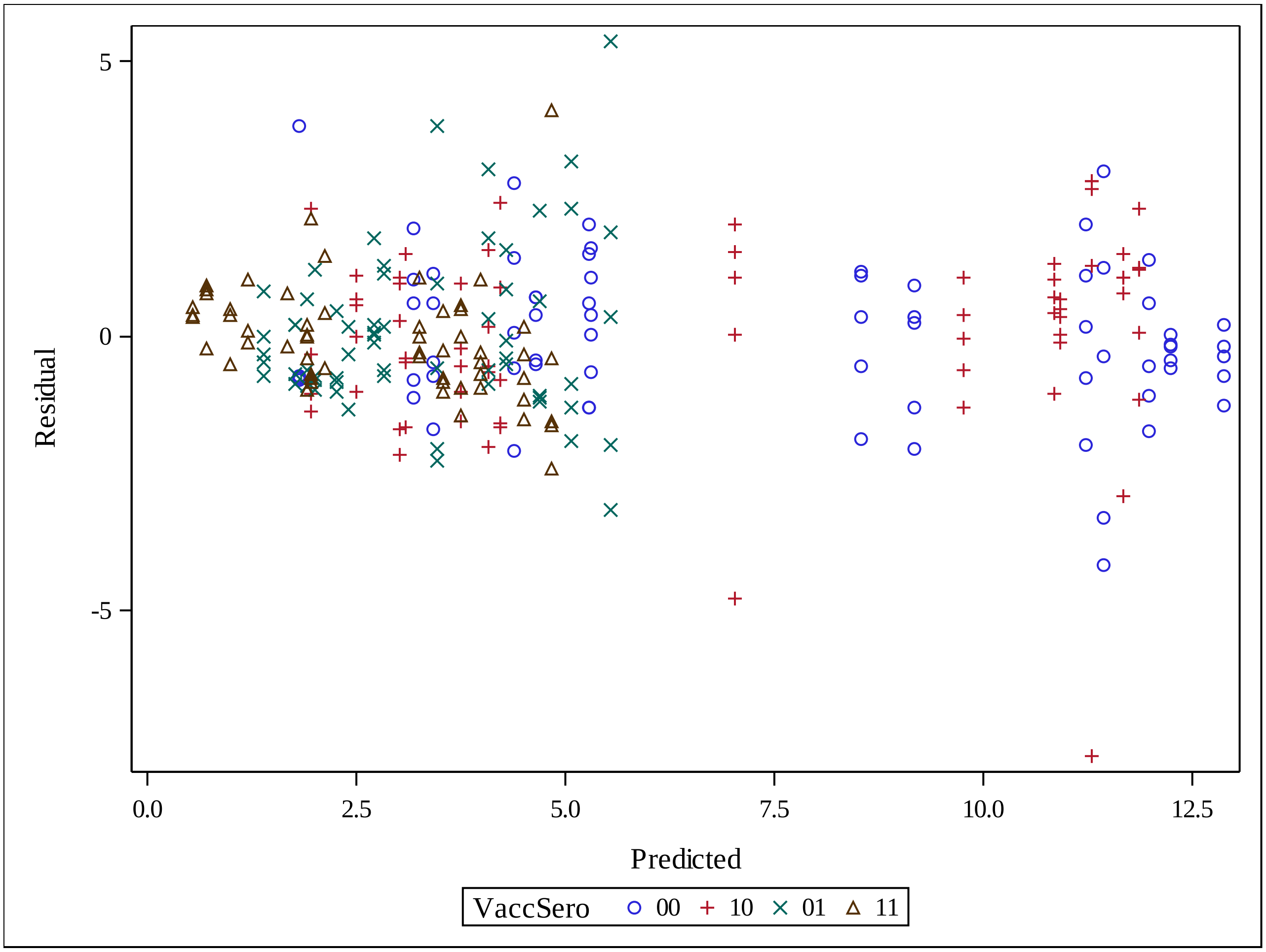

Residual
5
0
-5
0.0
2.5
5.0
7.5
10.0
12.5
Predicted
VaccSero
00
10
01
11

The MEANS Procedure

Analysis Variable : areaRect		
CamelID	N Obs	Sum
11	14	96.7460828
12	14	107.8487485
13	14	96.1011404
14	14	110.5697307
15	14	99.4420902
21	14	99.9070033
22	14	89.6964427
23	14	95.9023348
24	14	99.2099861
25	14	82.3156968
31	14	59.5459676
32	14	24.9573527
33	14	32.5058721
34	14	25.1188792
35	14	46.9222652
41	14	27.4368385
42	14	23.0595128
43	14	27.6492982
44	14	21.6483356
45	14	29.6137791

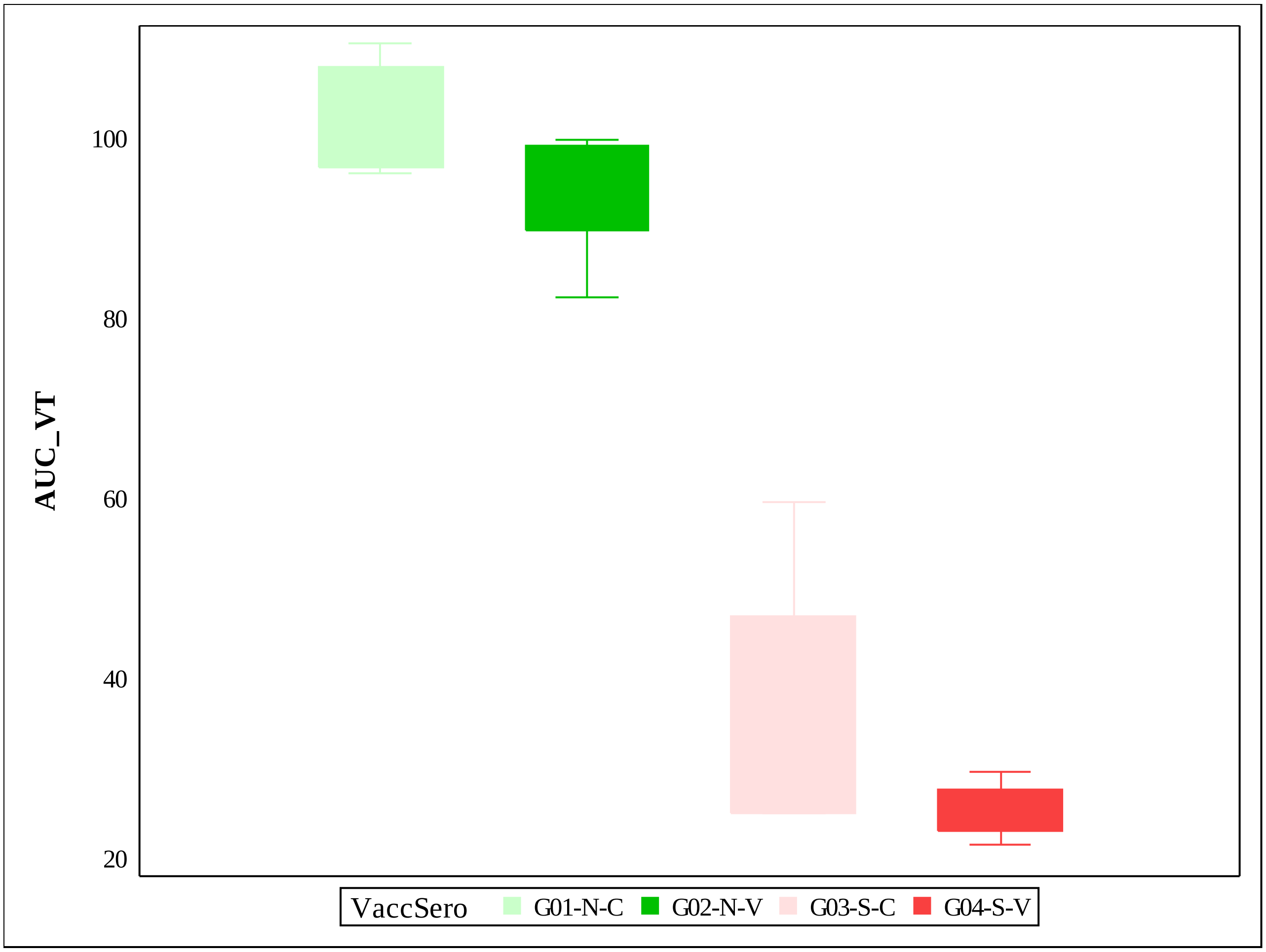
AUC_VT
100
80
60
40
20
VaccSero
G01-N-C
G02-N-V
G03-S-C
G04-S-V

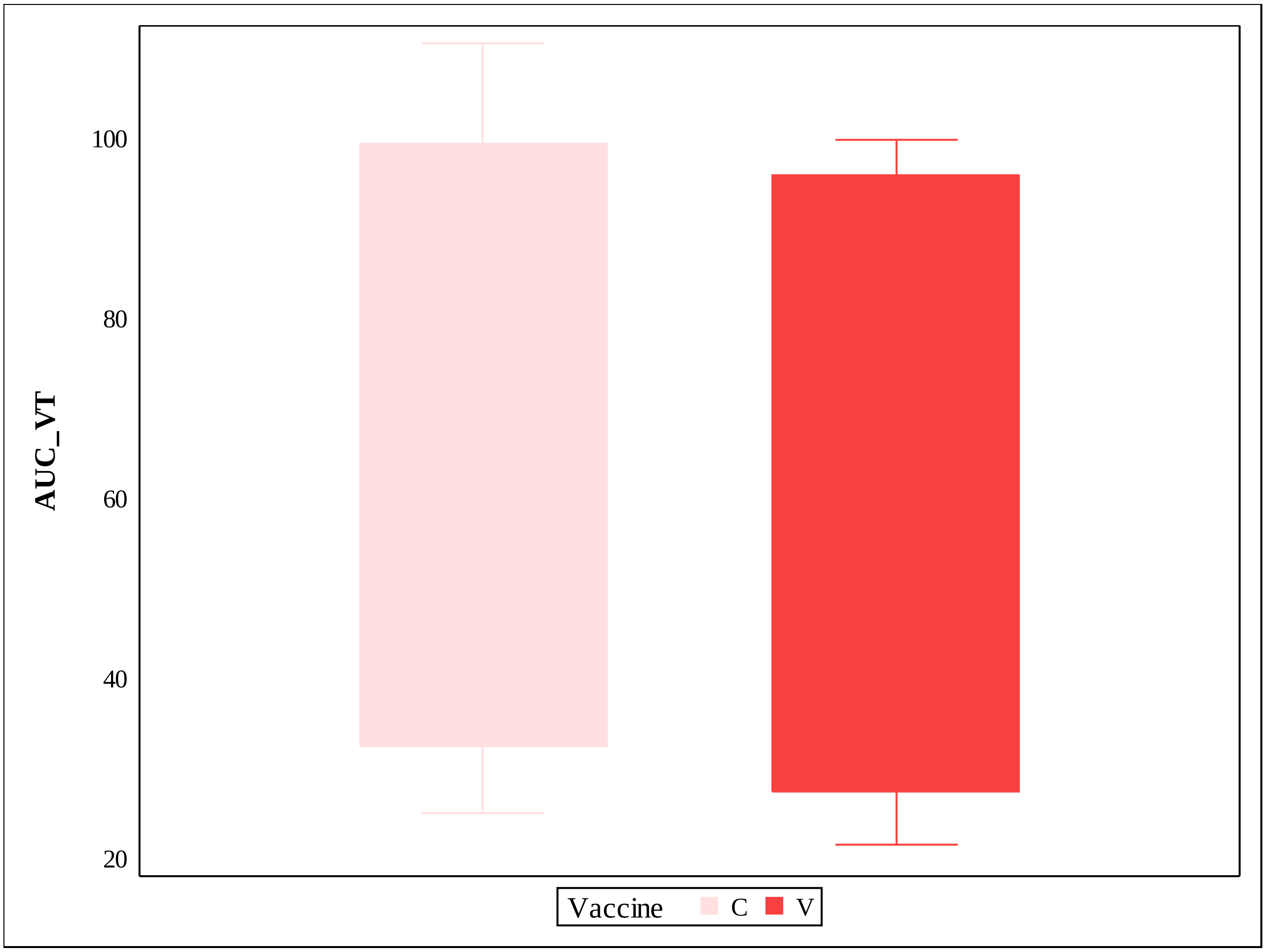
AUC_VT
100
80
60
40
20
Vaccine
C
V

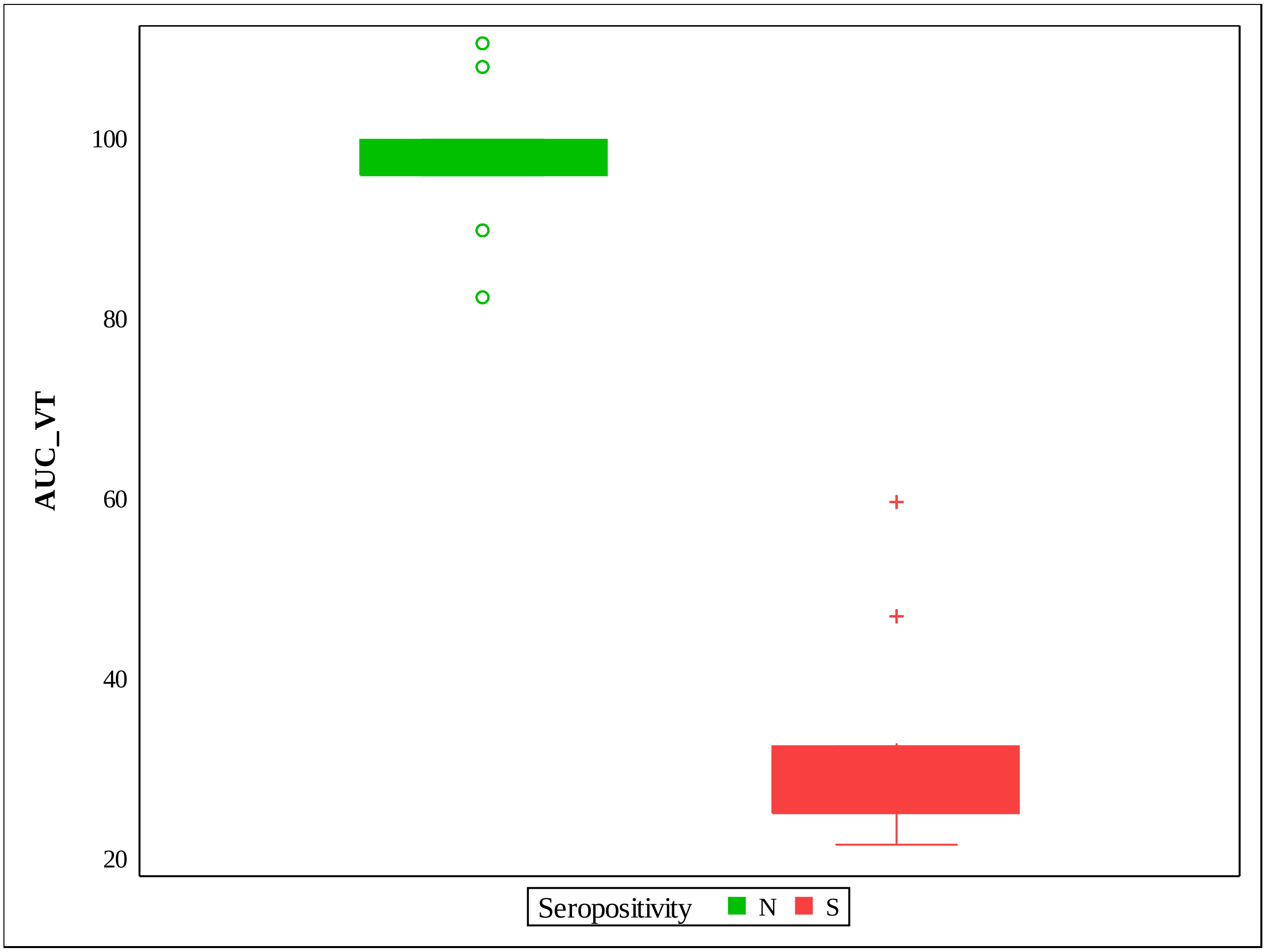
100
80
60
40
20
AUC_VT
Seropositivity
N
S

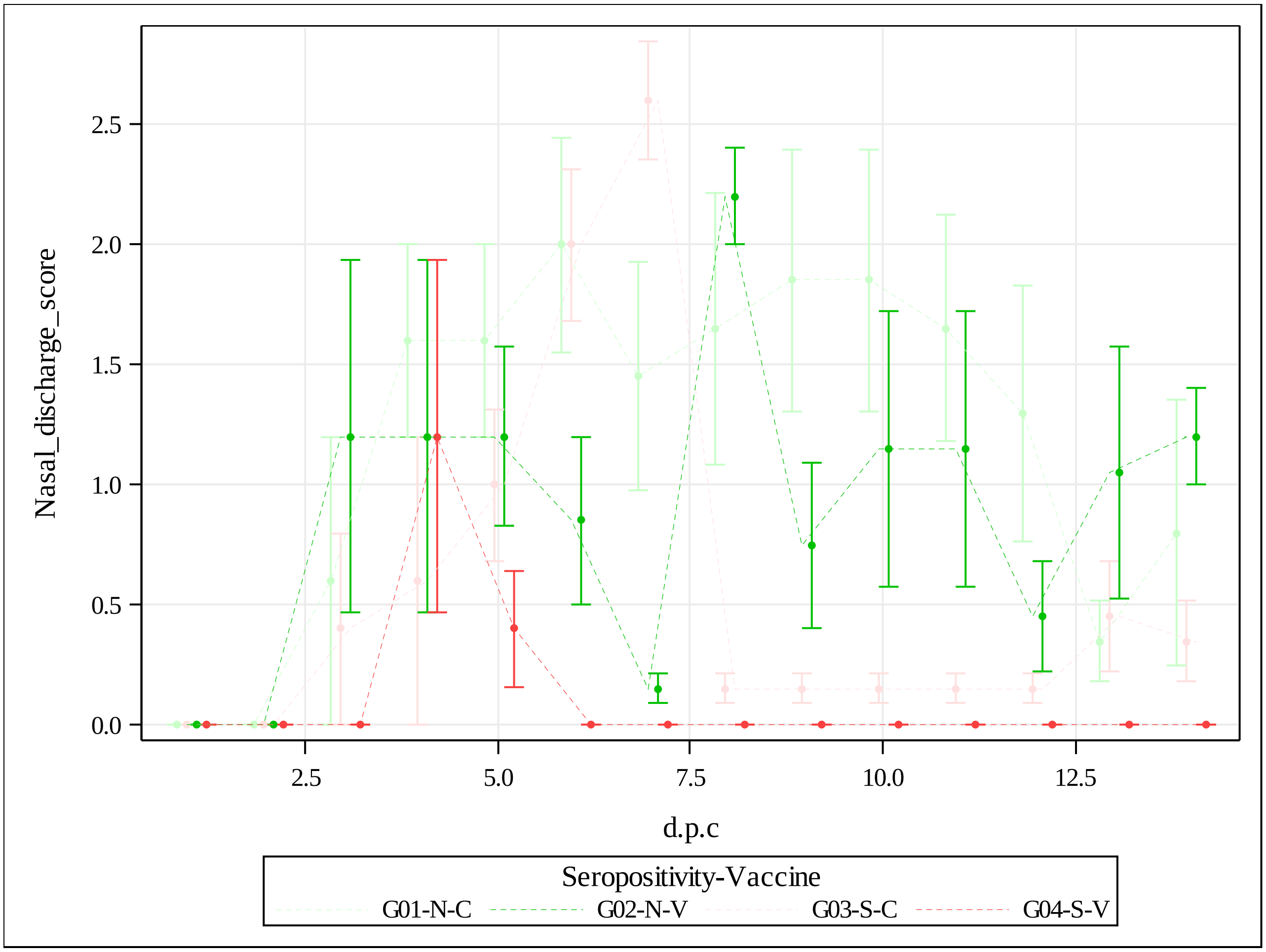

Nasal_discharge_score
0.0
0.5
1.0
1.5
2.0
2.5
2.5
5.0
7.5
10.0
12.5
d.p.c
Seropositivity-Vaccine
G01-N-C
G02-N-V
G03-S-C
G04-S-V

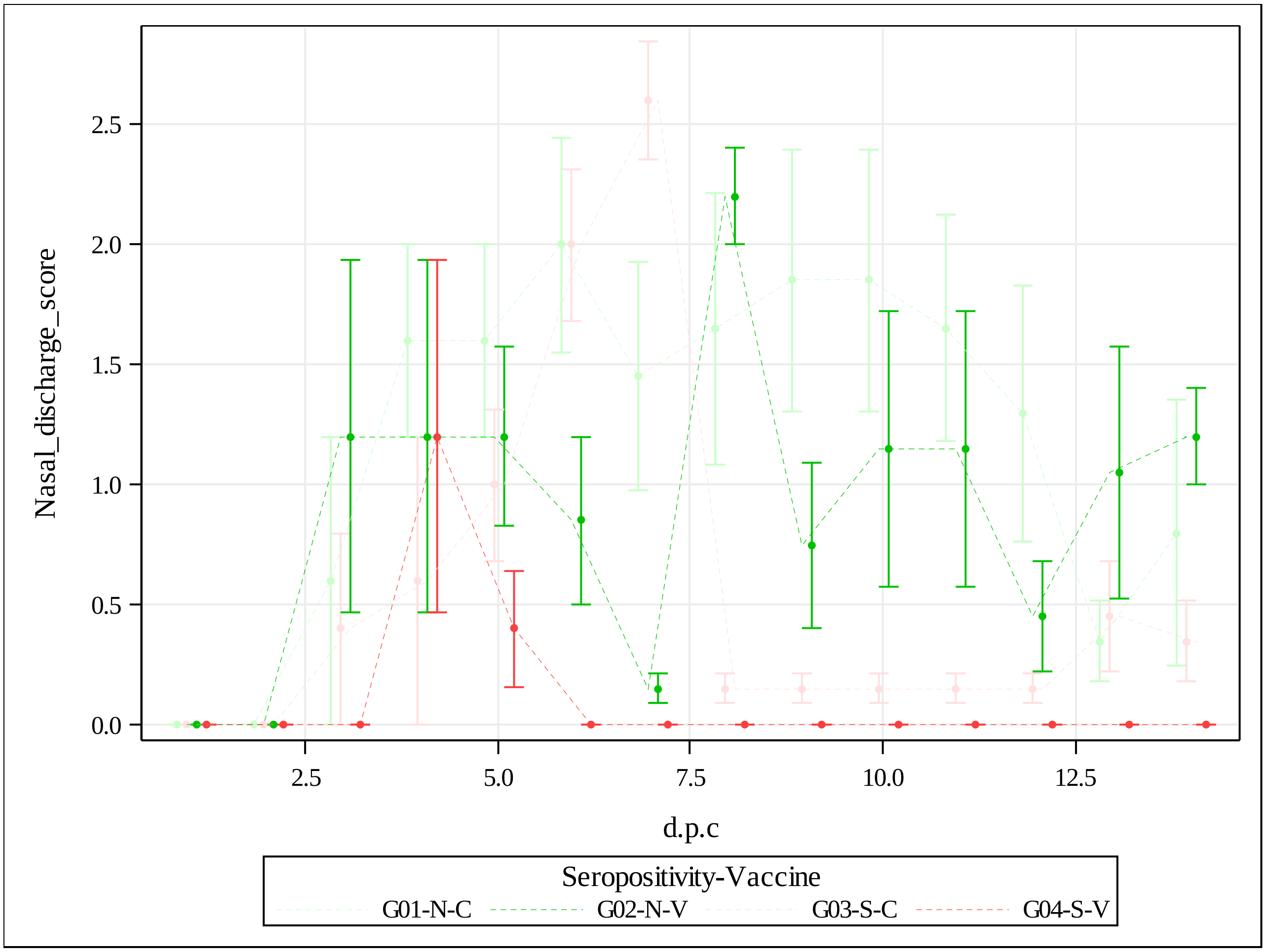
Nasal_discharge_score
0.0
0.5
1.0
1.5
2.0
2.5
2.5
5.0
7.5
10.0
12.5
d.p.c
Seropositivity-Vaccine
G01-N-C
G02-N-V
G03-S-C
G04-S-V

The Mixed Procedure

Model Information	
**Data Set**	WORK.DATAN0
**Dependent Variable**	Nasal_discharge_score
**Covariance Structure**	Autoregressive
**Subject Effect**	CamelID
**Estimation Method**	REML
**Residual Variance Method**	Profile
**Fixed Effects SE Method**	Model-Based
**Degrees of Freedom Method**	Between-Within

Class Level Information		
**Class**	**Levels**	**Values**
**d_p_c_**	14	1 2 3 4 5 6 7 8 9 10 11 12 13 14
**CamelID**	20	11 12 13 14 15 21 22 23 24 25 31 32 33 34 35 41 42 43 44 45
**Seropositivity**	2	0 1
**Vaccine**	2	0 1

Dimensions	
**Covariance Parameters**	2
**Columns in X**	75
**Columns in Z**	0
**Subjects**	20
**Max Obs Per Subject**	14

Number of Observations	
**Number of Observations Read**	280
**Number of Observations Used**	280
**Number of Observations Not Used**	0

Iteration History			
**Iteration**	**Evaluations**	**-2 Res Log Like**	**Criterion**
**0**	1	649.84733028	
**1**	2	569.06863698	0.00000262
**2**	1	569.06846431	0.00000000

The Mixed Procedure

Convergence criteria met.

Estimated R Matrix for CamelID 11														
**Row**	**Col1**	**Col2**	**Col3**	**Col4**	**Col5**	**Col6**	**Col7**	**Col8**	**Col9**	**Col10**	**Col11**	**Col12**	**Col13**	**Col14**
**1**	0.6028	0.3195	0.1694	0.08978	0.04759	0.02522	0.01337	0.007087	0.003756	0.001991	0.001055	0.000559	0.000297	0.000157
**2**	0.3195	0.6028	0.3195	0.1694	0.08978	0.04759	0.02522	0.01337	0.007087	0.003756	0.001991	0.001055	0.000559	0.000297
**3**	0.1694	0.3195	0.6028	0.3195	0.1694	0.08978	0.04759	0.02522	0.01337	0.007087	0.003756	0.001991	0.001055	0.000559
**4**	0.08978	0.1694	0.3195	0.6028	0.3195	0.1694	0.08978	0.04759	0.02522	0.01337	0.007087	0.003756	0.001991	0.001055
**5**	0.04759	0.08978	0.1694	0.3195	0.6028	0.3195	0.1694	0.08978	0.04759	0.02522	0.01337	0.007087	0.003756	0.001991
**6**	0.02522	0.04759	0.08978	0.1694	0.3195	0.6028	0.3195	0.1694	0.08978	0.04759	0.02522	0.01337	0.007087	0.003756
**7**	0.01337	0.02522	0.04759	0.08978	0.1694	0.3195	0.6028	0.3195	0.1694	0.08978	0.04759	0.02522	0.01337	0.007087
**8**	0.007087	0.01337	0.02522	0.04759	0.08978	0.1694	0.3195	0.6028	0.3195	0.1694	0.08978	0.04759	0.02522	0.01337
**9**	0.003756	0.007087	0.01337	0.02522	0.04759	0.08978	0.1694	0.3195	0.6028	0.3195	0.1694	0.08978	0.04759	0.02522
**10**	0.001991	0.003756	0.007087	0.01337	0.02522	0.04759	0.08978	0.1694	0.3195	0.6028	0.3195	0.1694	0.08978	0.04759
**11**	0.001055	0.001991	0.003756	0.007087	0.01337	0.02522	0.04759	0.08978	0.1694	0.3195	0.6028	0.3195	0.1694	0.08978
**12**	0.000559	0.001055	0.001991	0.003756	0.007087	0.01337	0.02522	0.04759	0.08978	0.1694	0.3195	0.6028	0.3195	0.1694
**13**	0.000297	0.000559	0.001055	0.001991	0.003756	0.007087	0.01337	0.02522	0.04759	0.08978	0.1694	0.3195	0.6028	0.3195
**14**	0.000157	0.000297	0.000559	0.001055	0.001991	0.003756	0.007087	0.01337	0.02522	0.04759	0.08978	0.1694	0.3195	0.6028

The Mixed Procedure

Estimated R Correlation Matrix for CamelID 11												
**Row**	**Col1**	**Col2**	**Col3**	**Col4**	**Col5**	**Col6**	**Col7**	**Col8**	**Col9**	**Col10**	**Col11**	**Col12**
**1**	1.0000	0.5301	0.2810	0.1489	0.07894	0.04184	0.02218	0.01176	0.006231	0.003303	0.001751	0.000928
**2**	0.5301	1.0000	0.5301	0.2810	0.1489	0.07894	0.04184	0.02218	0.01176	0.006231	0.003303	0.001751
**3**	0.2810	0.5301	1.0000	0.5301	0.2810	0.1489	0.07894	0.04184	0.02218	0.01176	0.006231	0.003303
**4**	0.1489	0.2810	0.5301	1.0000	0.5301	0.2810	0.1489	0.07894	0.04184	0.02218	0.01176	0.006231
**5**	0.07894	0.1489	0.2810	0.5301	1.0000	0.5301	0.2810	0.1489	0.07894	0.04184	0.02218	0.01176
**6**	0.04184	0.07894	0.1489	0.2810	0.5301	1.0000	0.5301	0.2810	0.1489	0.07894	0.04184	0.02218
**7**	0.02218	0.04184	0.07894	0.1489	0.2810	0.5301	1.0000	0.5301	0.2810	0.1489	0.07894	0.04184
**8**	0.01176	0.02218	0.04184	0.07894	0.1489	0.2810	0.5301	1.0000	0.5301	0.2810	0.1489	0.07894
**9**	0.006231	0.01176	0.02218	0.04184	0.07894	0.1489	0.2810	0.5301	1.0000	0.5301	0.2810	0.1489
**10**	0.003303	0.006231	0.01176	0.02218	0.04184	0.07894	0.1489	0.2810	0.5301	1.0000	0.5301	0.2810
**11**	0.001751	0.003303	0.006231	0.01176	0.02218	0.04184	0.07894	0.1489	0.2810	0.5301	1.0000	0.5301
**12**	0.000928	0.001751	0.003303	0.006231	0.01176	0.02218	0.04184	0.07894	0.1489	0.2810	0.5301	1.0000
**13**	0.000492	0.000928	0.001751	0.003303	0.006231	0.01176	0.02218	0.04184	0.07894	0.1489	0.2810	0.5301
**14**	0.000261	0.000492	0.000928	0.001751	0.003303	0.006231	0.01176	0.02218	0.04184	0.07894	0.1489	0.2810

Estimated R Correlation Matrix for CamelID 11		
**Row**	**Col13**	**Col14**
**1**	0.000492	0.000261
**2**	0.000928	0.000492
**3**	0.001751	0.000928
**4**	0.003303	0.001751
**5**	0.006231	0.003303
**6**	0.01176	0.006231
**7**	0.02218	0.01176
**8**	0.04184	0.02218
**9**	0.07894	0.04184
**10**	0.1489	0.07894
**11**	0.2810	0.1489
**12**	0.5301	0.2810
**13**	1.0000	0.5301
**14**	0.5301	1.0000

The Mixed Procedure

Covariance Parameter Estimates		
**Cov Parm**	**Subject**	**Estimate**
**AR(1)**	CamelID	0.5301
**Residual**		0.6028

Fit Statistics	
**-2 Res Log Likelihood**	569.1
**AIC (smaller is better)**	573.1
**AICC (smaller is better)**	573.1
**BIC (smaller is better)**	575.1

Null Model Likelihood Ratio Test		
**DF**	**Chi-Square**	**Pr > ChiSq**
1	80.78	<.0001

Solution for Fixed Effects								
**Effect**	**d_p_c_**	**Seropositivity**	**Vaccine**	**Estimate**	**Standard Error**	**DF**	**t Value**	**Pr > |t|**
**Intercept**				0.1875	0.3007	17	0.62	0.5412
**d_p_c_**	1			-0.1875	0.4252	221	-0.44	0.6597
**d_p_c_**	2			-0.1875	0.4252	221	-0.44	0.6596
**d_p_c_**	3			0.06250	0.4251	221	0.15	0.8832
**d_p_c_**	4			0.7625	0.4249	221	1.79	0.0741
**d_p_c_**	5			0.2625	0.4246	221	0.62	0.5370
**d_p_c_**	6			0.02500	0.4239	221	0.06	0.9530
**d_p_c_**	7			0.1375	0.4228	221	0.33	0.7453
**d_p_c_**	8			-0.01250	0.4205	221	-0.03	0.9763
**d_p_c_**	9			-0.4250	0.4163	221	-1.02	0.3084
**d_p_c_**	10			-0.3250	0.4081	221	-0.80	0.4267
**d_p_c_**	11			-0.2750	0.3923	221	-0.70	0.4841
**d_p_c_**	12			-0.3625	0.3606	221	-1.01	0.3159
**d_p_c_**	13			0.1000	0.2915	221	0.34	0.7319
**d_p_c_**	14			0	.	.	.	.
**Seropositivity**		0		0.8250	0.3472	17	2.38	0.0295

The Mixed Procedure

Solution for Fixed Effects								
**Effect**	**d_p_c_**	**Seropositivity**	**Vaccine**	**Estimate**	**Standard Error**	**DF**	**t Value**	**Pr > |t|**
**Seropositivity**		1		0	.	.	.	.
**Vaccine**			0	-0.02500	0.3472	17	-0.07	0.9434
**Vaccine**			1	0	.	.	.	.
**d_p_c_*Seropositivit**	1	0		-0.8250	0.4910	221	-1.68	0.0943
**d_p_c_*Seropositivit**	1	1		0	.	.	.	.
**d_p_c_*Seropositivit**	2	0		-0.8250	0.4909	221	-1.68	0.0943
**d_p_c_*Seropositivit**	2	1		0	.	.	.	.
**d_p_c_*Seropositivit**	3	0		-0.1250	0.4908	221	-0.25	0.7992
**d_p_c_*Seropositivit**	3	1		0	.	.	.	.
**d_p_c_*Seropositivit**	4	0		-0.3250	0.4906	221	-0.66	0.5084
**d_p_c_*Seropositivit**	4	1		0	.	.	.	.
**d_p_c_*Seropositivit**	5	0		-0.1250	0.4902	221	-0.25	0.7990
**d_p_c_*Seropositivit**	5	1		0	.	.	.	.
**d_p_c_*Seropositivit**	6	0		-0.4000	0.4895	221	-0.82	0.4147
**d_p_c_*Seropositivit**	6	1		0	.	.	.	.
**d_p_c_*Seropositivit**	7	0		-1.3250	0.4882	221	-2.71	0.0072
**d_p_c_*Seropositivit**	7	1		0	.	.	.	.
**d_p_c_*Seropositivit**	8	0		1.0250	0.4856	221	2.11	0.0359
**d_p_c_*Seropositivit**	8	1		0	.	.	.	.
**d_p_c_*Seropositivit**	9	0		0.4000	0.4807	221	0.83	0.4062
**d_p_c_*Seropositivit**	9	1		0	.	.	.	.
**d_p_c_*Seropositivit**	10	0		0.6000	0.4713	221	1.27	0.2043
**d_p_c_*Seropositivit**	10	1		0	.	.	.	.
**d_p_c_*Seropositivit**	11	0		0.5000	0.4530	221	1.10	0.2709
**d_p_c_*Seropositivit**	11	1		0	.	.	.	.
**d_p_c_*Seropositivit**	12	0		-0.02500	0.4164	221	-0.06	0.9522
**d_p_c_*Seropositivit**	12	1		0	.	.	.	.
**d_p_c_*Seropositivit**	13	0		-0.3500	0.3366	221	-1.04	0.2996
**d_p_c_*Seropositivit**	13	1		0	.	.	.	.
**d_p_c_*Seropositivit**	14	0		0	.	.	.	.
**d_p_c_*Seropositivit**	14	1		0	.	.	.	.
**d_p_c_*Vaccine**	1		0	0.02500	0.4910	221	0.05	0.9594
**d_p_c_*Vaccine**	1		1	0	.	.	.	.
**d_p_c_*Vaccine**	2		0	0.02500	0.4909	221	0.05	0.9594

The Mixed Procedure

Solution for Fixed Effects								
**Effect**	**d_p_c_**	**Seropositivity**	**Vaccine**	**Estimate**	**Standard Error**	**DF**	**t Value**	**Pr > |t|**
**d_p_c_*Vaccine**	2		1	0	.	.	.	.
**d_p_c_*Vaccine**	3		0	-0.07500	0.4908	221	-0.15	0.8787
**d_p_c_*Vaccine**	3		1	0	.	.	.	.
**d_p_c_*Vaccine**	4		0	-0.07500	0.4906	221	-0.15	0.8786
**d_p_c_*Vaccine**	4		1	0	.	.	.	.
**d_p_c_*Vaccine**	5		0	0.5250	0.4902	221	1.07	0.2854
**d_p_c_*Vaccine**	5		1	0	.	.	.	.
**d_p_c_*Vaccine**	6		0	1.6000	0.4895	221	3.27	0.0013
**d_p_c_*Vaccine**	6		1	0	.	.	.	.
**d_p_c_*Vaccine**	7		0	1.9750	0.4882	221	4.05	<.0001
**d_p_c_*Vaccine**	7		1	0	.	.	.	.
**d_p_c_*Vaccine**	8		0	-0.1750	0.4856	221	-0.36	0.7189
**d_p_c_*Vaccine**	8		1	0	.	.	.	.
**d_p_c_*Vaccine**	9		0	0.6500	0.4807	221	1.35	0.1777
**d_p_c_*Vaccine**	9		1	0	.	.	.	.
**d_p_c_*Vaccine**	10		0	0.4500	0.4713	221	0.95	0.3407
**d_p_c_*Vaccine**	10		1	0	.	.	.	.
**d_p_c_*Vaccine**	11		0	0.3500	0.4530	221	0.77	0.4406
**d_p_c_*Vaccine**	11		1	0	.	.	.	.
**d_p_c_*Vaccine**	12		0	0.5250	0.4164	221	1.26	0.2087
**d_p_c_*Vaccine**	12		1	0	.	.	.	.
**d_p_c_*Vaccine**	13		0	-0.1000	0.3366	221	-0.30	0.7667
**d_p_c_*Vaccine**	13		1	0	.	.	.	.
**d_p_c_*Vaccine**	14		0	0	.	.	.	.
**d_p_c_*Vaccine**	14		1	0	.	.	.	.

Type 3 Tests of Fixed Effects				
**Effect**	**Num DF**	**Den DF**	**F Value**	**Pr > F**
**d_p_c_**	13	221	3.65	<.0001
**Seropositivity**	1	17	19.33	0.0004
**Vaccine**	1	17	5.82	0.0274
**d_p_c_*Seropositivit**	13	221	4.86	<.0001
**d_p_c_*Vaccine**	13	221	4.64	<.0001

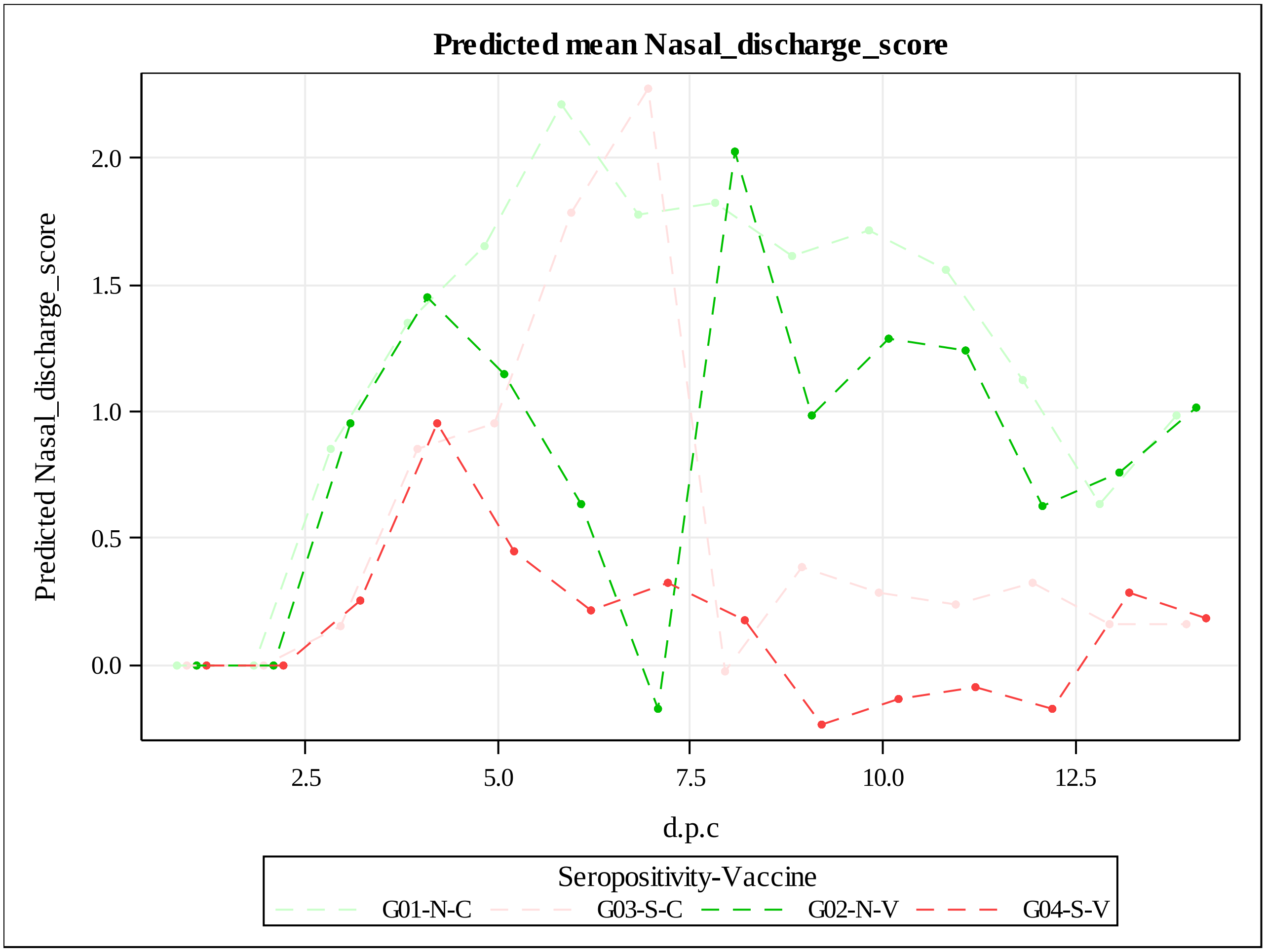
Predicted mean Nasal_discharge_score
Predicted Nasal_discharge_score
0.0
0.5
1.0
1.5
2.0
2.5
5.0
7.5
10.0
12.5
d.p.c
Seropositivity-Vaccine
G01-N-C
G03-S-C
G02-N-V
G04-S-V

Predicted mean Nasal_discharge_score

The MEANS Procedure

Analysis Variable : areaRect		
**CamelID**	**N Obs**	**Sum**
11	14	11.6250000
12	14	14.3750000
13	14	29.1250000
14	14	21.7500000
15	14	4.6250000
21	14	8.7500000
22	14	23.5000000
23	14	17.0000000
24	14	4.7500000
25	14	5.7500000
31	14	4.3750000
32	14	8.0000000
33	14	7.7500000
34	14	12.3750000
35	14	7.3750000
41	14	0
42	14	0
43	14	4.0000000
44	14	0
45	14	4.0000000

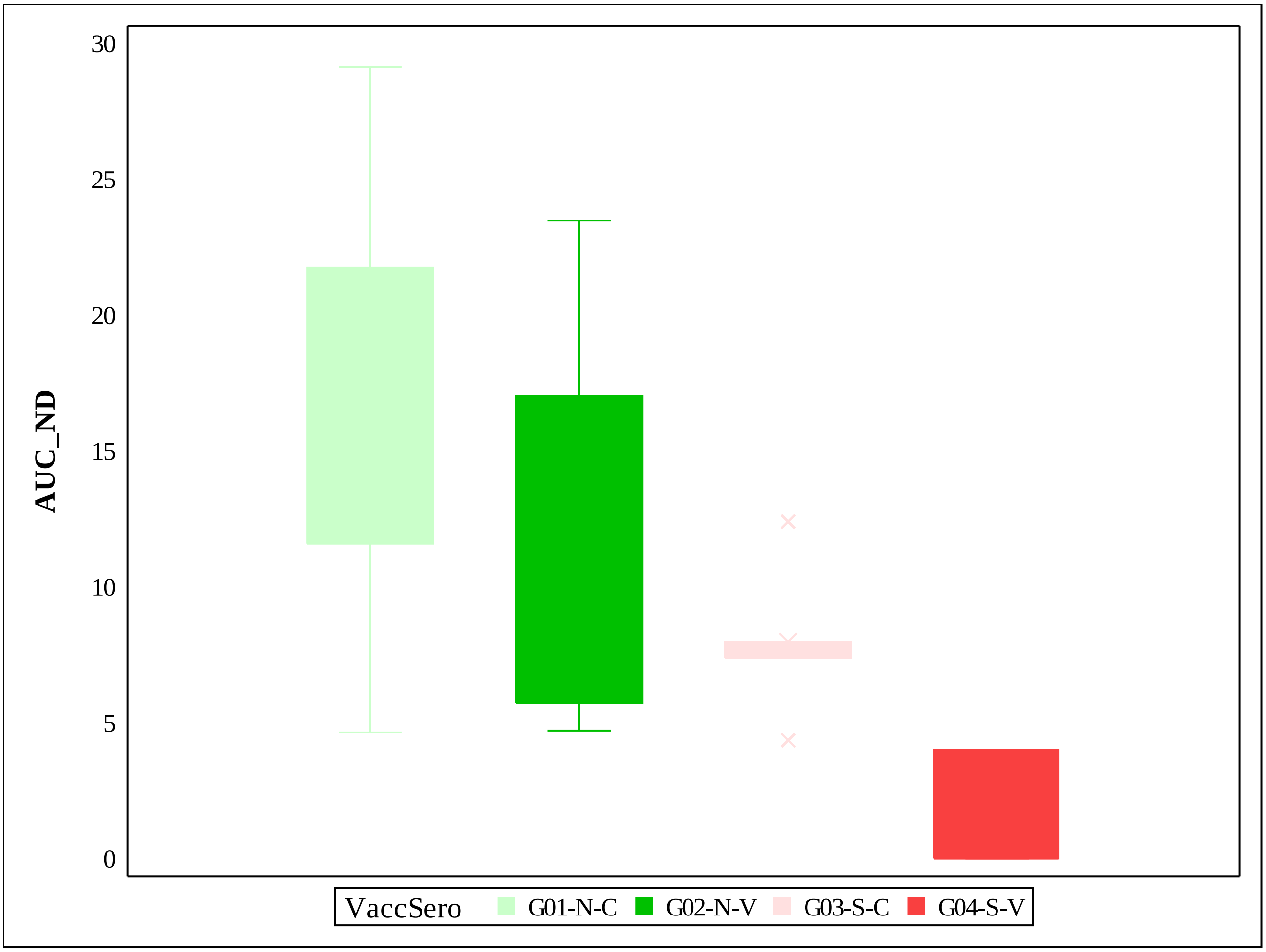
AUC_ND
30
25
20
15
10
5
0
VaccSero
G01-N-C
G02-N-V
G03-S-C
G04-S-V

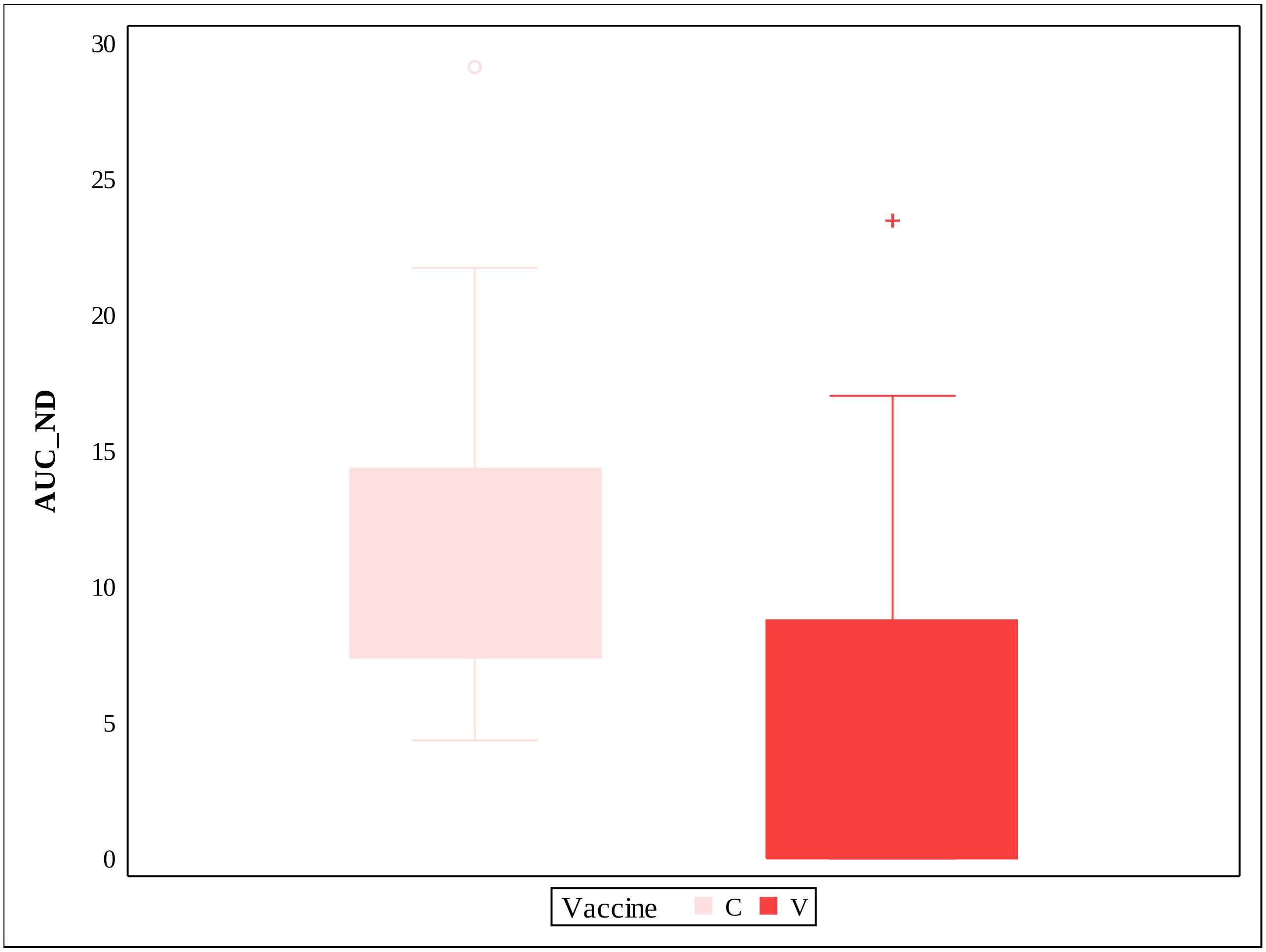
30
25
20
15
10
5
0
AUC_ND
Vaccine
C
V

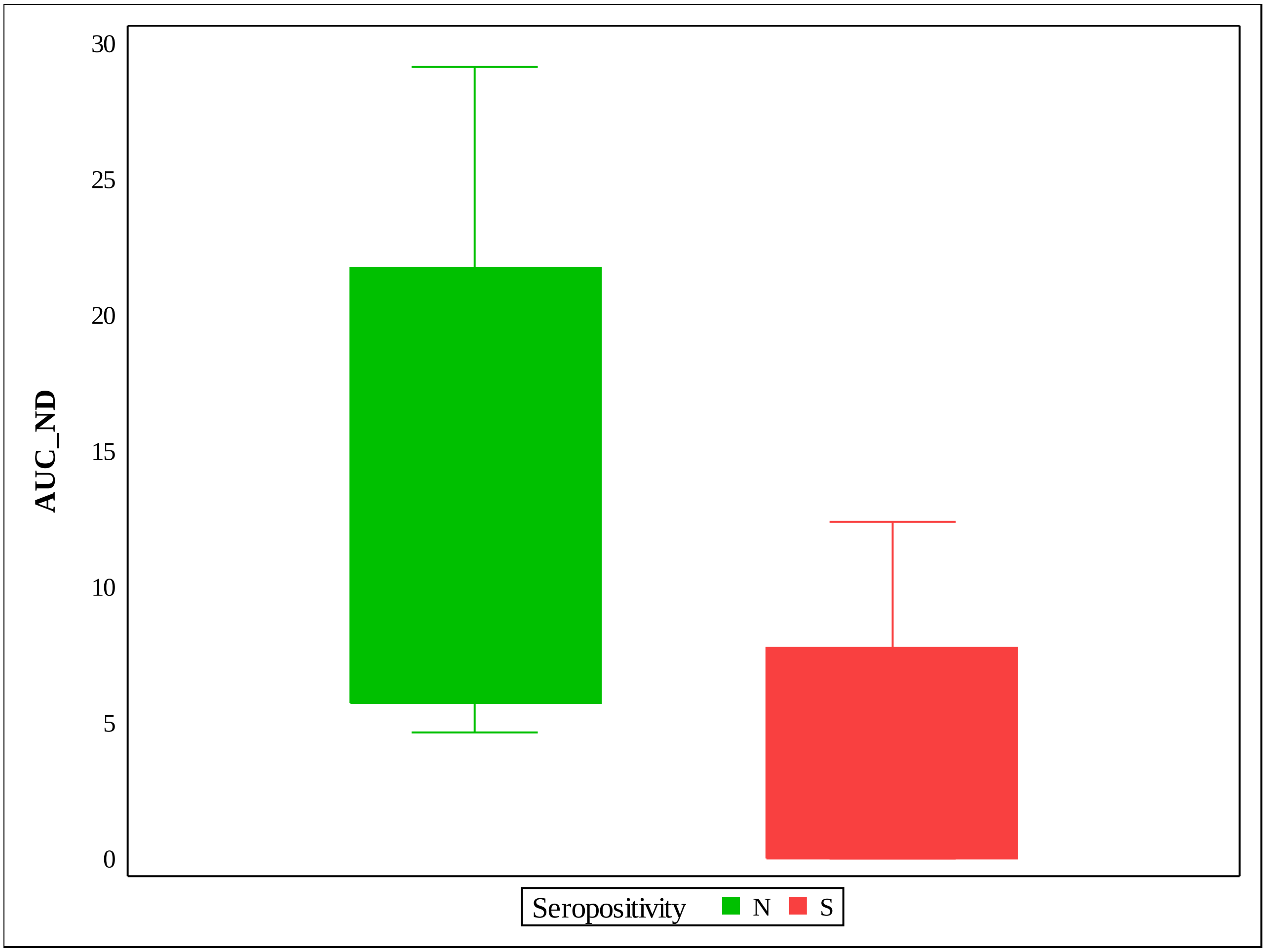
30
25
20
15
10
5
0
AUC_ND
Seropositivity
N
S

The GLM Procedure

Class Level Information		
**Class**	**Levels**	**Values**
**Seropositivity**	2	N S
**Vaccine**	2	C V

**Number of Observations Read**	20
**Number of Observations Used**	20

The GLM Procedure

Dependent Variable: AUC_VT

Source	DF	Sum of Squares	Mean Square	F Value	Pr > F
**Model**	3	22279.06791	7426.35597	87.97	<.0001
**Error**	16	1350.65958	84.41622		
**Corrected Total**	19	23629.72749			

R-Square	Coeff Var	Root MSE	AUC_VT Mean
0.942841	14.17659	9.187830	64.80987

Source	DF	Type I SS	Mean Square	F Value	Pr > F
**Seropositivity**	1	21732.58209	21732.58209	257.45	<.0001
**Vaccine**	1	533.73977	533.73977	6.32	0.0230
**Seropositivi*Vaccine**	1	12.74605	12.74605	0.15	0.7027

Source	DF	Type III SS	Mean Square	F Value	Pr > F
**Seropositivity**	1	21732.58209	21732.58209	257.45	<.0001
**Vaccine**	1	533.73977	533.73977	6.32	0.0230
**Seropositivi*Vaccine**	1	12.74605	12.74605	0.15	0.7027

The GLM Procedure

Dependent Variable: AUC_VT

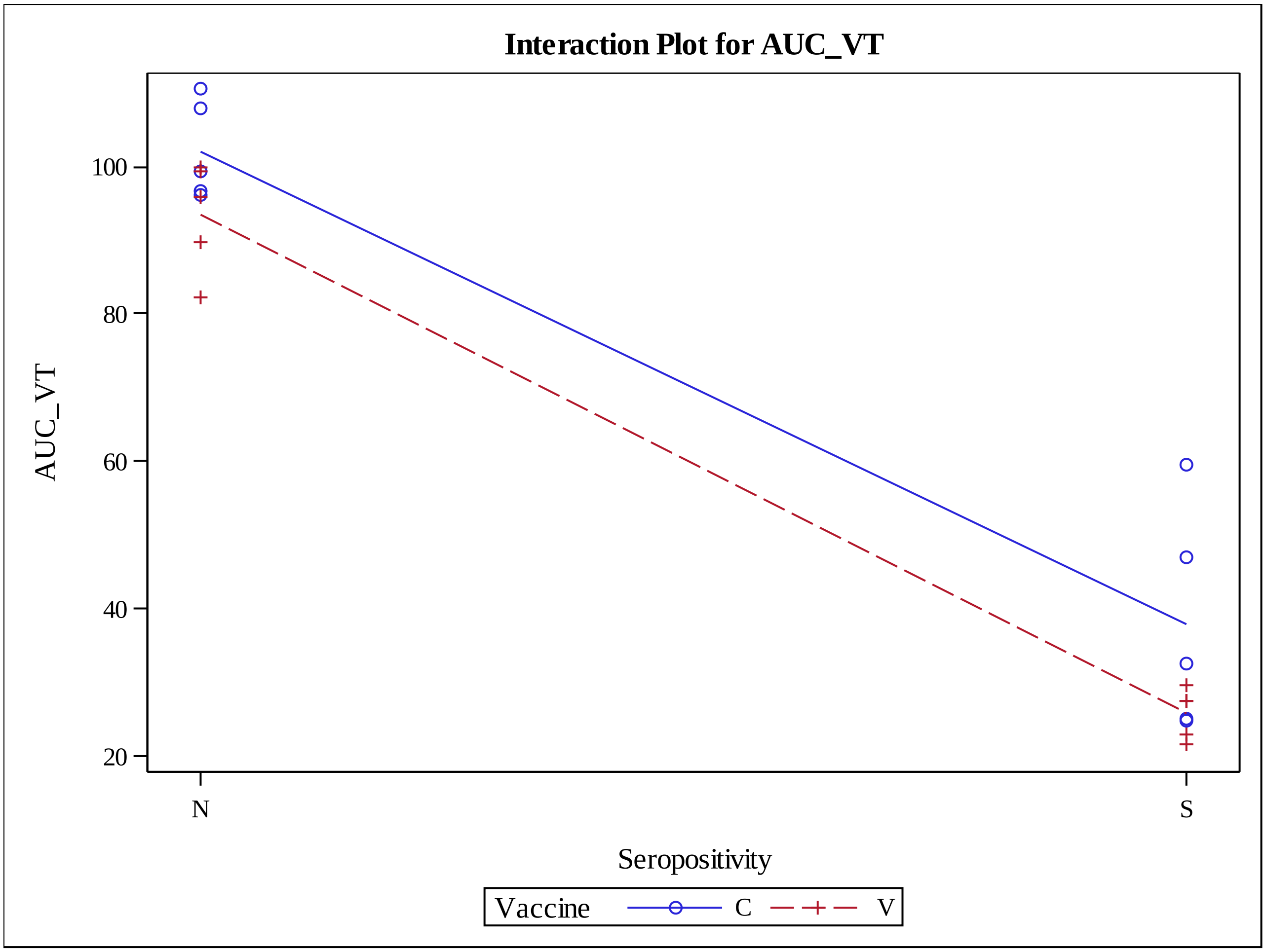

The GLM Procedure

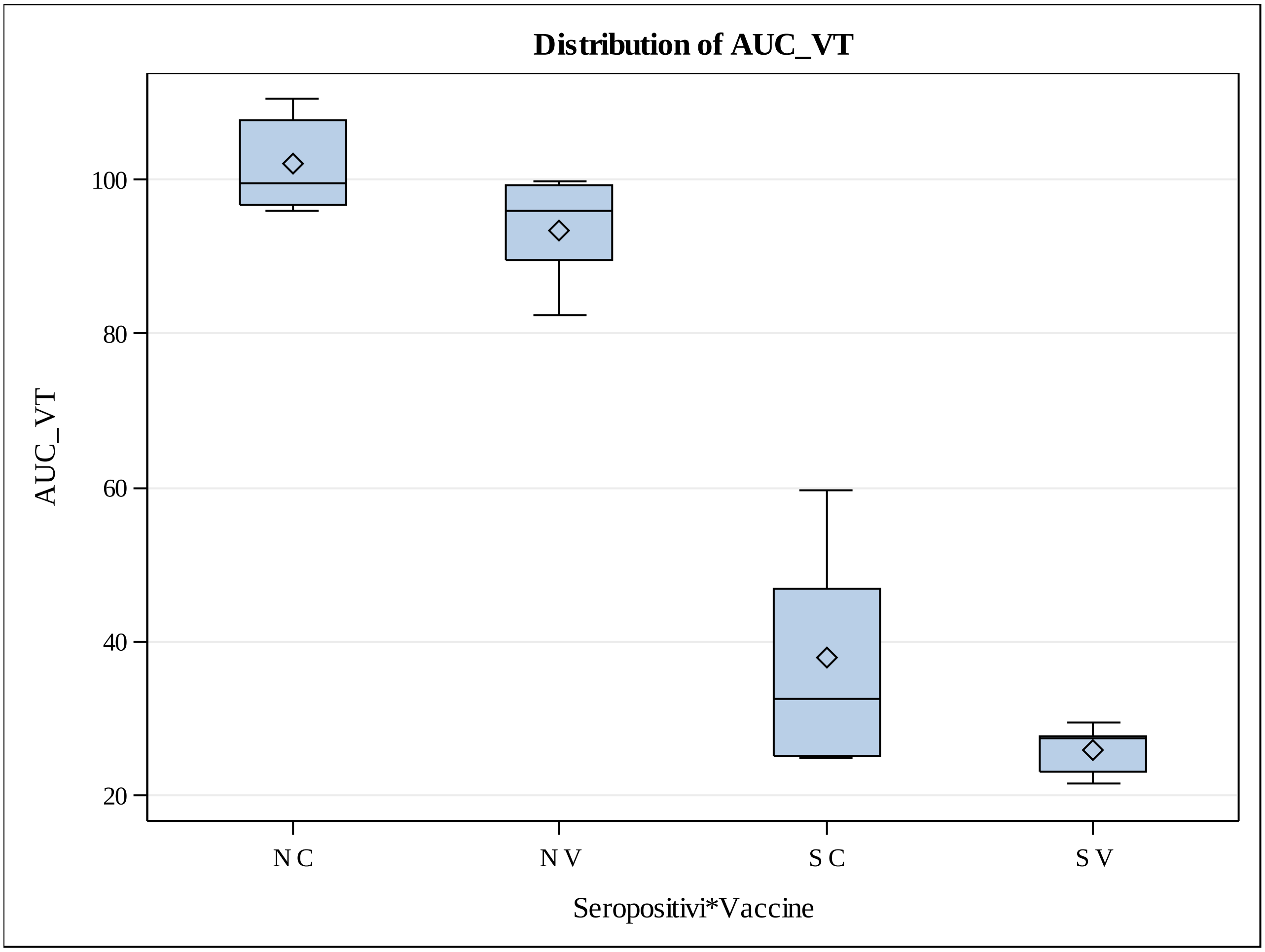

Level of Seropositivity	Level of Vaccine	N	AUC_VT Mean	AUC_VT Std Dev
**N**	**C**	5	102.141559	6.6424966
**N**	**V**	5	93.406293	7.3982536
**S**	**C**	5	37.810067	15.0820632
**S**	**V**	5	25.881553	3.3673947

The GLM Procedure

Class Level Information		
**Class**	**Levels**	**Values**
**Seropositivity**	2	N S
**Vaccine**	2	C V

**Number of Observations Read**	20
**Number of Observations Used**	20

The GLM Procedure

Source	DF	Sum of Squares	Mean Square	F Value	Pr > F
**Model**	3	584.852344	194.950781	4.67	0.0157
**Error**	16	667.406250	41.712891		
**Corrected Total**	19	1252.258594			

R-Square	Coeff Var	Root MSE	AUC_ND Mean
0.467038	68.29929	6.458552	9.456250

Source	DF	Type I SS	Mean Square	F Value	Pr > F
**Seropositivity**	1	435.9445313	435.9445313	10.45	0.0052
**Vaccine**	1	143.7820313	143.7820313	3.45	0.0819
**Seropositivi*Vaccine**	1	5.1257813	5.1257813	0.12	0.7305

Source	DF	Type III SS	Mean Square	F Value	Pr > F
**Seropositivity**	1	435.9445313	435.9445313	10.45	0.0052
**Vaccine**	1	143.7820313	143.7820313	3.45	0.0819
**Seropositivi*Vaccine**	1	5.1257813	5.1257813	0.12	0.7305

The GLM Procedure

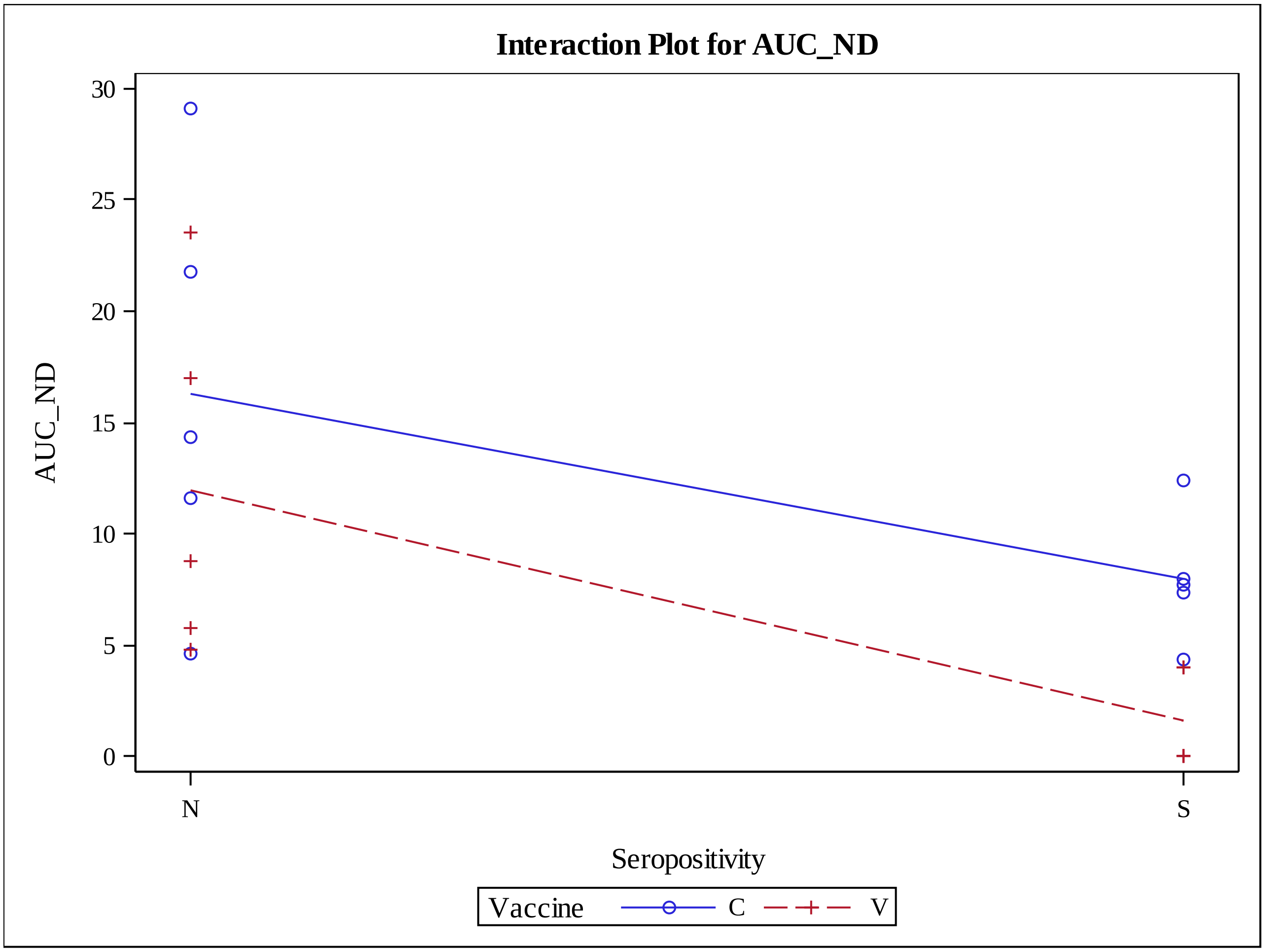

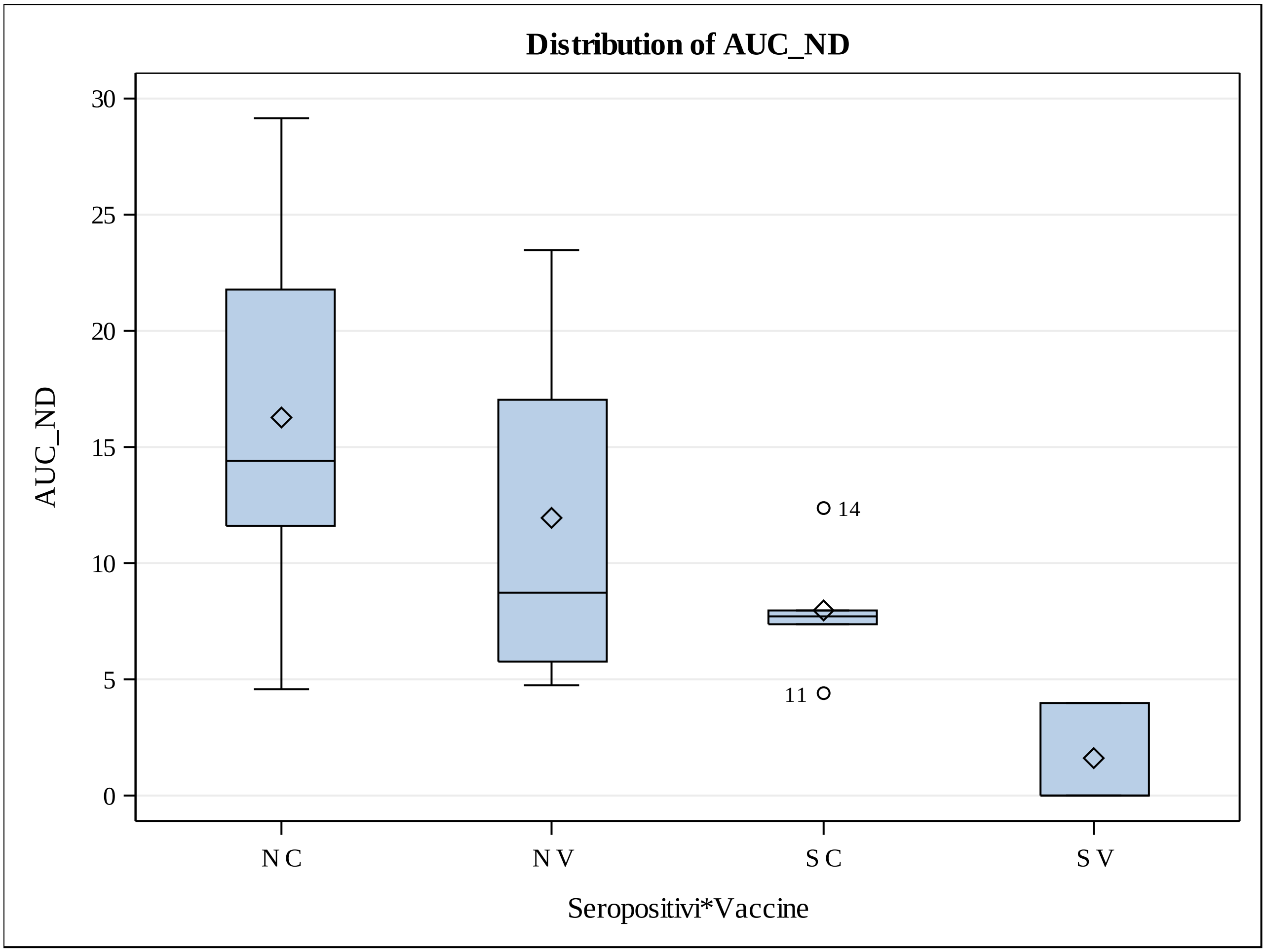

Level of Seropositivity	Level of Vaccine	N	AUC_ND	
			Mean	Std Dev
**N**	**C**	5	16.3000000	9.43464361
**N**	**V**	5	11.9500000	8.05333782
**S**	**C**	5	7.9750000	2.86056157
**S**	**V**	5	1.6000000	2.19089023

Table 1: Cohen's f^2 the effect size of virus titre post challenge by grouping factors and day post challenge.

Cohen's f^2	Grouping factor	Day post challenge
0.128	Seropositivity	1
1.714	Seropositivity	2
4.055	Seropositivity	3
11.069	Seropositivity	4
4.787	Seropositivity	5
9.636	Seropositivity	6
8.912	Seropositivity	7
1.805	Seropositivity	8
1.623	Seropositivity	9
2.916	Seropositivity	10
0.826	Seropositivity	11
2.957	Seropositivity	12
0.621	Seropositivity	13
0.002	Seropositivity	14
0.321	Vaccine	1
0.186	Vaccine	2
0.013	Vaccine	3
0.018	Vaccine	4
0.364	Vaccine	5
0.324	Vaccine	6
0.446	Vaccine	7
0.165	Vaccine	8
0.315	Vaccine	9
0.060	Vaccine	10
0.341	Vaccine	11
0.827	Vaccine	12
0.016	Vaccine	13
0.001	Vaccine	14

Table 2: Cohen's f^2 the effect size of nasal discharge score by grouping factors and day post challenge

Cohen's *f*2	Grouping factor	Day post challenge
	Seropositivity	1
	Seropositivity	2
0.109	Seropositivity	3
0.038	Seropositivity	4
0.265	Seropositivity	5
0.097	Seropositivity	6

0.157	Seropositivity	7
2.156	Seropositivity	8
0.787	Seropositivity	9
0.782	Seropositivity	10
0.780	Seropositivity	11
0.432	Seropositivity	12
0.128	Seropositivity	13
0.419	Seropositivity	14
	Vaccine	1
	Vaccine	2
0.002	Vaccine	3
0.002	Vaccine	4
0.135	Vaccine	5
1.326	Vaccine	6
2.395	Vaccine	7
0.025	Vaccine	8
0.205	Vaccine	9
0.070	Vaccine	10
0.047	Vaccine	11
0.169	Vaccine	12
0.009	Vaccine	13
0.000	Vaccine	14

Cohen's f^2 is used to measure the practical significance of our test, we report the local effect size Cohen's f^2 for repeated-measures[1].

Reference:

1- Selya, A. S., Rose, J. S., Dierker, L. C., Hedeker, D., & Mermelstein, R. J. (2012). A Practical Guide to Calculating Cohen's f(2), a Measure of Local Effect Size, from PROC MIXED. *Frontiers in psychology*, *3*, 111. doi:10.3389/fpsyg.2012.00111
